# Construction of a Bacterial Lipidomics Analytical Platform: Pilot Validation with Bovine Paratuberculosis Serum

**DOI:** 10.3390/metabo13070809

**Published:** 2023-06-29

**Authors:** Paul L. Wood, Erdal Erol

**Affiliations:** 1 Metabolomics Unit, College of Veterinary Medicine, Lincoln Memorial University, 6965 Cumberland Gap Pkwy, Harrogate, TN 37752, USA; 2 Department of Veterinary Science, Veterinary Diagnostic Laboratory, University of Kentucky, Lexington, KY 40546, USA; erdal.erol@uky.edu

**Keywords:** bacterial lipidomics, bovine paratuberculosis, mycolic acids, lipoteichoic acids, high-resolution mass spectrometry

## Abstract

Lipidomics analyses of bacteria offer the potential to detect and monitor infections in a host since many bacterial lipids are not present in mammals. To evaluate this omics approach, we first built a database of bacterial lipids for representative Gram-positive and Gram-negative bacteria. Our lipidomics analysis of the reference bacteria involved high-resolution mass spectrometry and electrospray ionization with less than a 1.0 ppm mass error. The lipidomics profiles of bacterial cultures clearly distinguished between Gram-positive and Gram-negative bacteria. In the case of bovine paratuberculosis (PTB) serum, we monitored two unique bacterial lipids that we also monitored in *Mycobacterium avian* subspecies *PTB*. These were PDIM-B C82, a phthiodiolone dimycocerosate, and the trehalose monomycolate hTMM 28:1, constituents of the bacterial cell envelope in mycolic-containing bacteria. The next step will be to determine if lipidomics can detect subclinical PTB infections which can last 2-to-4 years in bovine PTB. Our data further suggest that it will be worthwhile to continue building our bacterial lipidomics database and investigate the further utility of this approach in other infections of veterinary and human clinical interest.

## 1. Introduction

Lipidomics is a rapidly evolving “omics” platform that provides valuable information regarding structural, energy source/reserve, and signal-transduction lipid pools. Bacteria possess a number of unique lipids that are not present in their mammalian hosts. This provides the opportunity of lipidomics to obtain valuable non-mammalian lipid data that can (i) detect bacterial infection in a host, (ii) monitor the progression of an infection, (iii) monitor the efficacy of treatments on an infection, and (iv) potentially define new targets in the design of targeted antimicrobial therapeutics.

While the individual lipids of a given lipid family for a bacterial strain will alter with development and with environmental stresses, lipid families will be preserved and can be monitored. Our first high-level overview is a comparison of our current knowledge base for Gram-positive vs. Gram-negative bacterial lipidomics.

### 1.1. Gram-Positive Bacteria

#### 1.1.1. Gram-Positive Bacteria: Lipoteichoic Acids

Gram-positive bacteria possess a cytoplasmic membrane and a multilaminar cell wall [1]. Between the cell membrane and cell wall is a heteropolysaccharide meshwork of peptidoglycans and arabinogalactans. Teichoic acids, which anchor to peptidoglycans in the cell wall, and lipoteichoic acids (LTAs), which are found in the cell membrane, are lipids that are unique to Gram-positive bacteria, providing a strong negative charge to the cell wall [2]. Precursors to LTAs that have been monitored in Gram-positive bacteria include a number of glycolipids (Table 1).

The diversity of LTA precursor lipidomes between different bacterial species is demonstrated by the detection of DHMG in only 12 of 19 clostridia species examined [9]. The further modification of these lipids through the addition of phosphoethanolamine only was present in 4 of those 12 species [9].

#### 1.1.2. Gram-Positive Bacteria: Modified Phosphatidylglycerols

Aminoacylation of phosphatidylglycerol (PG) is another unique feature in the lipidome of Gram-positive bacteria (Table 2). The pathway for these aminoacylations is phosphatidic acid → CDP-DG → phosphatidylglycerophosphate → PG → aminoacyl-PG.

The diversity of amino acyl lipidomes between different bacterial species is demonstrated by the detection of lysyl-PG in only 5 of 24 clostridia species examined [9] and the detection of alanyl-PG in only 3 of 24 clostridia species examined [9].

#### 1.1.3. Gram-Positive Bacteria: Mycolic Acids

A very unique family of glycolipids is also present in the outer wall of a number of bacteria in the Actinomycetes taxonomic group. These are the mycolic acids present in mycolic-acid-containing bacteria (MACB) [1,27] which include Mycobacteria (*M. tuberculosis*, *M. leprae*, *M. bovis*, *Tskamurella pulmonis*, *Rhodococcus erythropolis*, *R. opacus*, and *R. equi*) and Corynebacteria (*C. glutamicum*) [27,28]. Long-chain mycolic acids are covalently bound in the inner layer of the cell wall but are present as free acids in the outer domain. Lipids in this lipid family are diverse (Table 3).

#### 1.1.4. Gram-Positive Bacteria: Mannosyl Phosphoinositols (PIMs)

PIMs are unique to Mycobacteria (*M. tuberculosis*, *M. leprae*, *M. bovis*, *Tskamurella pulmonis*, *Rhodococcus erythropolis*, and *R. opacus*, *R.equi*) and Corynebacteria (*C. glutamicum*) [27,28,29] (Table 4). They are critical structural components of both the outer and inner membranes of the cell envelope.

#### 1.1.5. Gram-Positive Bacteria: Aminoacyl Lipids

While bacteria possess low levels of choline (PC) and ethanolamine (PE) glycerophospholipids, a number of aminoacylated forms of these lipids are present in the membranes of Gram-positive bacteria (Table 5).

### 1.2. Gram-Negative Bacteria

#### 1.2.1. Gram-Negative Bacteria: Glycosyl Hydroxy Fatty Acids (HFAs) and Glycosyl-FAHFAs

Gram-negative bacteria possess a cell envelope comprising an inner and outer membrane with an intermediate peptidoglycan layer. Lipid A is a major membrane lipid in Gram-negative bacteria. This complex lipid has a core scaffold of P-glucosamine-glucosamine with acyl or FAHFA substitutions of the nitrogen in each hexose and acyl substitution of the hydroxy group in P-glucosamine [46,47,48].

Fatty acyls of hydroxy fatty acids (FAHFAs) [49] are present at high concentrations in Gram-negative bacteria, and both the glycosylated and aminoacyl forms are critical membrane constituents. The glycosylation of hydroxy fatty acids yields rhamnolipids, which act as biosurfactant antimicrobials. Representative glycolipids in Gram-negative bacteria are presented in Table 6.

#### 1.2.2. Gram-Negative Bacteria: Aminoacyl Hydroxy Fatty Acids (HFAs) and FAHFAs

Gram-negative bacteria possess a diverse array of aminoacyl HFAs and FAHFAs that serve as virulence factors (Table 7).

#### 1.2.3. Gram-Negative Bacteria: Modified Ceramides

Gram-negative bacteria possess several unique modified ceramides which are considered to contribute to membrane charge (Table 8).

#### 1.2.4. Gram-Negative Bacteria: Glycosyl-Glycerophosphoalkylamines

Several complex glycolipids have been identified as regulators of cell temperature in *Thermus thermophilus* [91,92]: PLGN (Diacyl-PA-Acyl-Alkylamine-Glucosamine) and PGL (Diacyl-PA-Acyl-Alkylamine-N-Acetyl-Glucosamine).

#### 1.2.5. Gram-Negative Bacteria: Sterols

Gram-negative bacteria utilize several unique cholesteryl acyl-glycosides as immunostimulants and hopanoids which order membrane lipids and regulate membrane permeability [97] (Table 9).

#### 1.2.6. Gram-Negative Bacteria: Secondary Metabolites

Gram-negative bacteria produce a number of secondary metabolites that they utilize to protect against other microbes (Table 10).

In summary, the wide diversity of bacterial lipids offers the potential to differentiate different bacterial species via lipidomics analyses. For example, previous studies of polar lipids in *Clostridia* spp. in four different groups of bacteria based on morphological and biochemical criteria demonstrated that three of the four groups possessed lipids that distinguished each group. All groups had high levels of PE and PG. However, Group I (*C. sporogenes* prototype) possessed PE-NAcGlu-DGs, Group II (*C. butyricum* prototype) possessed glycerol and PG acetals of ethanolamine plasmalogens, Group III (*C. novyi* prototype) possessed aminoacyl-PGs, and Group IV (*C. subterminale* prototype) had no distinguishing polar lipids [106,107]. Extending future lipidomics analyses across a broader scope than just polar lipids should further increase our ability to differentiate ongoing bacterial infections.

The objective of our study was to initiate building a bacterial lipidomics database that we could utilize to interrogate serum from cows infected with paratuberculosis and provide the groundwork required to continue building and expanding the database such that it will allow for the interrogation of other clinically relevant infections.

## 2. Materials and Methods

### 2.1. Bacterial Processing

Bacterial pellets purchased from the ATTC (Manassas, VA, USA) were sonicated (Thermo Fisher FB50) in 1 mL of methanol and 1 mL of water containing 2 nanomoles of [^13^C_3_]DG 36:2 (Larodan, Monroe, MI, USA). Next 2 mL of tert-butylmethylether was added, and the samples were shaken at room temperature for 30 min (Thermo Fisher Multitube Vortexer, Waltham, MS, USA). Next, the samples were centrifuged at 4000× *g* for 30 min at room temperature. From the upper organic layer of these centrifuged samples, 1 mL aliquots were transferred to a deep-well microplate. The microplate samples were dried via vacuum centrifugation (Eppendorf Vacfuge Plus, Hamburg, Germany).

The Gram-positive bacterial pellets which we evaluated were *Mycobacterium avium*, ss. Paratuberculosis (ATCC 700535), *Staphylococcus aureus* (ATCC 10832), *Mycobacterium bovis* (ATTC 35737), *Mycobacterium smegmatis* (ATCC 14468), *Rhodococcus equi* (ATCC 7699), *Enterococcus faecalis* (ATCC 19433), and *Corynebacterium glutamicum* (13032). The purchased Gram-negative bacterial pellets were *Helicobacter pylori* (ATCC 43504), *Pseudomonas aeruginosa* (ATCC 10145), *Proteus mirabilis* (ATCC 12453), *Moraxella bovoculi* (ATCC BBA-1259), and *Escherichia coli* (ATCC 12435).

### 2.2. Lipidomics Analysis

We utilized published data and lipid databases for bacterial lipids and then incorporated them into our established lipidomics analytical platform [106,108,109,110,111,112] such that now we can interrogate approximately 11,000 individual lipids. As a pilot to evaluate the utility of this platform to detect active bacterial infections, we utilized the platform to examine the lipidome of a number of representative Gram-positive and Gram-negative bacteria and plasma samples from cows with paratuberculosis [112].

Specifically, to the dried samples, we added 200 μL of 2-propanol:methanol:chloroform (8:4:4), containing 5 mM ammonium chloride [108,111]. Lipids were characterized by flow infusion analysis (FIA) with electrospray ionization (ESI). FIA at 20 µL/minute was performed utilizing high-resolution (140,000 at 200 amu) data acquisition with an orbitrap mass spectrometer (Thermo Q Exactive) [106,108,109,110,111,112]. The FIA included a 30 s scan in the positive ESI mode (300–1500 amu), followed by a 30 s scan in the negative ESI mode (290–1500 amu). Between sample injections, the syringe and tubing were flushed with 1 mL of methanol, followed by 1 mL of hexane: ethyl acetate: chloroform: water (3:2:1:0.1). FIA has the advantages of high sample throughput with a short analysis time for each sample and data acquisition with a constant concentration of the lipid matrix.

For MS/MS analyses, parent ions were selected with a 0.4 amu window and collision energies of 15, 30, and 50 arbitrary units. Product ions were monitored with a resolution of 240,000. Product ions with a <1.0 ppm mass error are listed in Supplementary Table S3. We utilized Lincoln Memorial University, Metabolomics Unit, Flow Infusion Lipidomics Analytical Platform (Version 1.0).

### 2.3. Bovine PTB Serum Samples

Serum samples (100 μL) from our previous research [112] were used for this study and processed as described above. The cattle (n = 10) were 2-to-2.5-year-old angus. PTB infection was confirmed utilizing enzyme-linked immunosorbent assay (ELISA) (IDEXX MAP ELISA Ab Test kit, Westbrood, ME, USA). All testing was performed at the University of Kentucky Veterinary Diagnostic Laboratory (UKVDL), a fully accredited laboratory of the American Association of Veterinary Laboratory Diagnosticians (AAVLD).

### 2.4. Data Reduction

To our established in-house lipid database in Excel (Microsoft 365), we added the exact masses for a large number of individual bacterial lipids. Exact masses were obtained from online databases and the published literature. The databases we used included LipidMaps [PMID 33037133], *E. coli* Metabolome Database (ECMDB) [PMID 26481353], Yeast Metabolome Database (YMDB) [PMID 27899612], Mycobacterium tuberculosis Database (Mtb LipidDB) [PMID 21285232], Chemical Entities of Biological Interest (ChEBI) [PMID 26467479], Human Metabolome Database (HMDB) [PMID 34986597], Seaweed Metabolite Database (SWMD) [PMID 21423723], PubChem [PMID 33151290], and PubMed [PMID 33085945].

Mass spectrometric data were imported into this spreadsheet. This included individual scanned masses and their associated peak intensities. Based on the infusion solvent, the predominant ions were [M+H]^+^ or [M+NH_4_]^+^ in positive electrospray ionization (PESI), and they were [M-H]^−^ or [M+Cl]^−^ in NESI [108,111]. To define which ions were optimal for different lipid families, along with defining MS/MS criteria for structural validation, we purchased a number of microbial lipid standards. This included mycolic acids (Cat. 791280 and 791282), acyl-ceramides (Cat. 860626), lysyl-PG (Cat. 840521), sulfogalactosyl-ceramides (Cat. 860571), monogalactosyl-DG (Cat. 840523), and digalactosyl-DG (Cat. 840524) from Avanti Polar Lipids (Alabaster, AL, USA) and lipid A variants (Cat. SML-2430, Cat, L6895, and L5399), acyl trehalose (Cat. 30564), trehalose dimycolates (Cat. T3034), and rhamnolipids (Cat. R95MD and R95DD) from Sigma-Aldrich (St. Louis, MO, USA) to gain practical experience. In the case of lipid classes for which analytical standards were not available, we utilized the experiences from the prior literature and our in-house experience with our infusion solvent.

For each lipid in the Excel mass list, the imported data were searched for a matching mass with <1.0 ppm mass error. For positive hits, the extracted mass and the associated peak intensity were imported into a new active spreadsheet. The specific details for each lipid class, along with the associated ionization modes and MS/MS products, are presented in Supplementary Table S3, which details all the lipid classes included in our lipidomics analytical platform, along with citations for representative publications.

## 3. Results

### 3.1. Gram-Positive Bacteria

#### 3.1.1. *M. avium* Specific Lipids: Phthiodiolone Dimycocerosates and Diacyltrehaloses

**Figure 1:**
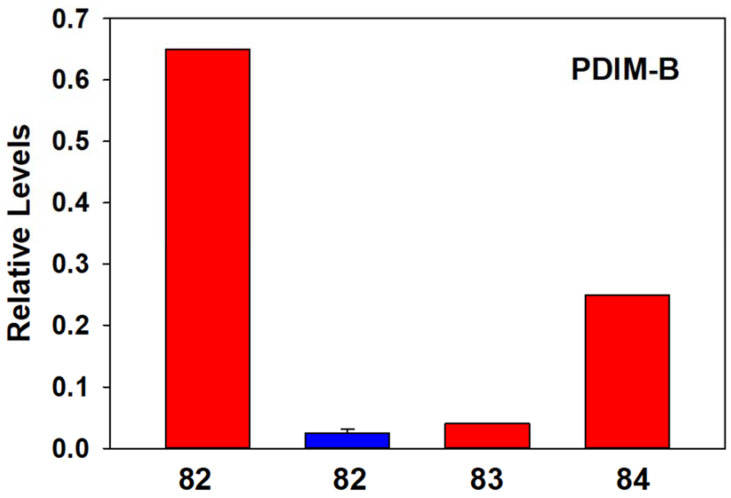
Relative PDIM-B levels in the bacterial pellet of *M. avium* (Red bars) and in the serum of 10 cows infected with PTB (blue bar; mean ± SD).

*M. avium* was unique in that it was the only Gram-positive species we examined that possessed phthiodiolone dimycocerosates and diacyltrehaloses (Figure 1 and Supplementary Table S1). The phthiodiolone dimycocerosates (PDIMs) are long-chain β-diols esterified at the hydroxy groups with multimethyl-branched fatty acids (mycocerosic acids). We specifically monitored PDIM-B forms in which a position 2 of the diol is a keto group. The dominant member of this lipid family was PDIM-B C82 in the ATCC bacterial pellets and was detected in the serum of cattle with paratuberculosis but not in control cows (Figure 1 and Supplementary Table S1). In contrast, while we detected diacyltrehaloses in the *M. avium* bacterial pellet (Supplementary Table S1), these lipids were undetectable in the serum of infected cows. The diacyltrehaloses were in the DAT2 family which have a fatty acid (16:0 to 19:0) and a mycolipanolic fatty acid substituent. The mycolipanolic fatty acids were 3-hydroxy-2,4,6-methyl fatty acids of 24 to 28 carbons.

#### 3.1.2. Trehalose Mycolates

**Figure 2:**
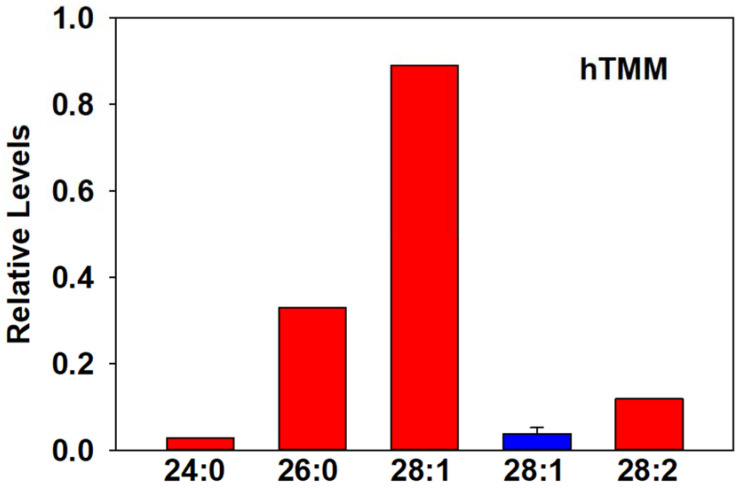
Relative hTMM levels in the bacterial pellet of *M. avium* (Red bars) and in the serum of 10 cows infected with PTB (blue bar; mean ± SD).

Hydroxy-trehalose monomycolates (hTMMs) were monitored in all of the examined bacteria except for *S. aureus* and *E. faecalis* (Supplementary Table S1). Each bacterial strain had a different dominant hTMM. In the case of *M. avium*, hTMM 28:1 was the dominant member of the lipid family and was also detected in the serum of PTB-positive cattle (Figure 2). While acetylTMMs were monitored in *M. avium* and a number of other Gram-positive bacteria (Supplementary Table S1), we did not detect any of this lipid family in the serum of infected cows.

#### 3.1.3. Lipoteichoic Acid Precursors

Lipoteichoic acid precursors (LTAPs; dihexosyldiacylglycerol-glycerol phosphate), along with the mono-alanine and di-alanine analogs, were not detected in the *M. avium* bacterial pellet (Supplementary Table S1). LTAP 32:0 was monitored in *S. aureus*, while LTAPs and Ala-LTAPs were monitored in the bacterial pellets from *R. equi*, *E. faecalis*, and *C. glutamicum*. As with other lipids, the dominant LTAP lipid family member was different for each bacterial strain. Di-Ala-LTAPs were detected only in *R. equi* bacterial pellets.

#### 3.1.4. Mannosyl Phosphoinositols (PIM1)

Acyl-PIM1 family members were only monitored in the *C. glutamicum* bacterial pellets (Supplementary Table S1), consistent with prior studies [16]. The acyl-PIM1 family has also been reported for a number of *Mycobacteria* [41]; however, we did not detect any acyl-PIM1 in the *Mycobacteria* we studied. This may have resulted from low levels and/or ion suppression.

#### 3.1.5. Mycolic Acids

**Figure 3:**
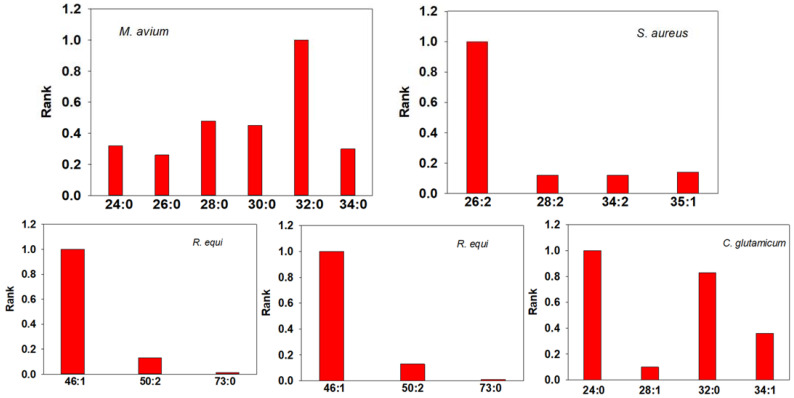
Bacterial mycolic acid levels presented as a rank order for *M. avium*, *S. aureus*, *R. equi*, *E. faecalis*, and *C. glutamicum*. The data are presented in Supplementary Table S1. With FIA, we cannot distinguish between double bonds and cyclopropane groups in the mycolic acids designated as unsaturated.

**Figure 4:**
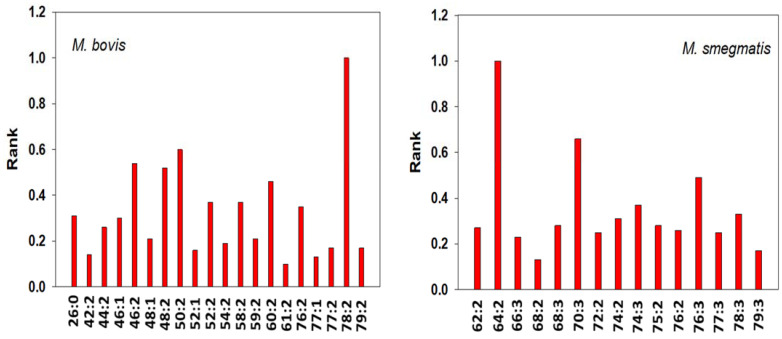
Bacterial mycolic acid levels presented as a rank order for *M. bovis* and *M. smegmatis*. The data are presented in Supplementary Table S1. With FIA, we cannot distinguish between double bonds and cyclopropane groups in the mycolic acids designated as unsaturated.

All of the Gram-positive bacteria that we studied were found to contain mycolic acids (Figure 3 and Figure 4; Supplementary Table S1). A diverse array of mycolic acids was monitored in the bacterial pellets. Most mycolic acids are tethered in the outer membrane, but there are small membrane levels of free mycolic acids [31,32,33,34,113], as demonstrated in Figure 3 and Figure 4. For the unsaturated lipids, our data do not distinguish between a double bond or a cyclopropyl substitution [113]. Both *M. bovis* and *M. smegmatis* mycolic acids were skewed to a distribution of longer-chain fatty acyl substituents (Figure 4). Interestingly, only these two bacterial strains had measurable levels of epoxymycolic acids (Supplementary Table S1). It also needs to be noted that our analyses do not distinguish between the isobars of oxygenated lipids [113]. For example, epoxymycolic acid 77:1 = ketomycolic acid 77:1 = methoxymycolic acid 77:2.

Dicarboxylic mycolic acids were only detected in *M. avium* and *M. bovis* (Supplementary Table S1).

The complexity of mycolic acids in bacteria was reflected in our analysis of the serum from cows infected with PTB. Four of the ten cows had levels of mycolic acid 50:2 (0.0011 ± 0.00064), five cows had dicarboxylic acid 82:1 (0.0053 ± 0.00065), two cows had dicarboxylic mycolic acid 84:1, one cow had dicarboxylic mycolic acid 82:2, and one cow had dicarboxylic mycolic acid 84:2. These lipids were not detected in the 10 control cows. This heterogeneity of detectable mycolic acids in the serum of infected cows may be reflective of different stages of the PTB infection, which is known to progress slowly over time [114].

#### 3.1.6. Glycopeptidolipids (GPLs)

The cell walls of a number of *Mycobacteria* contain a family of unique GPLs that consist of a hydroxy fatty acid coupled to a peptide which in turn is coupled to rhamnose [115,116,117]. The hydroxy fatty acid has a deoxytalose (dTal) glycation which has 0-to-2 possible acetylations. The peptide is Phe-Thr-Ala-Alaninol, and the terminal rhamnose has 0-to-3 possible O-methylations. This lipid family serves as cell-surface antigens.

We monitored an array of GPLs with the rank order of prevalence *C. glutamicum* > *M. smegmatis* > *R. equi* > *M. bovis* (Supplementary Table S1).

#### 3.1.7. Sulfonolipids

Sulfonolipids are characterized by the replacement of serine in the sphingolipid base by the sulfonic acid capnine generating sulfobacins (monohydroxy) and sulfocristamides (di-hydroxy) [118]. These lipids are required for gliding motility and demonstrate pro-inflammatory and cytotoxic activities [118]. Both *C. glutamicum* and *M. bovis* were found to possess these highly charged sphingolipids (Supplementary Table S1).

#### 3.1.8. Alpha-Acyl Hydroxy Fatty Acids (AAHFAs)

AAHFAs are a unique family of FAHFA lipids in which case the acylation is at a hydroxy group on carbon 2, with the acyl substitution being butyric acid [119]. The functions of these newly discovered lipids remain to be elaborated. In our analyses, we found high levels of AAHFAs in *M. avium* and moderate levels in *S. aureus* and *M. bovis* (Supplementary Table S1).

### 3.2. Gram-Negative Bacteria

Gram-negative bacteria lack the cell wall characteristic of Gram-positive bacteria. Lipid A is a major membrane lipid in the cell envelope, comprising an inner and outer membrane with an intermediate peptidoglycan layer. While intact lipid A molecules are large and tethered, a number of lipid A precursors are easily analyzed via conventional lipid-extraction procedures. Modified fatty acyls of hydroxy fatty acids (FAHFAs) are one example of these lipid A constituents that are absent from Gram-positive bacteria.

#### 3.2.1. Aminoacyl FAHFAs

**Figure 5:**
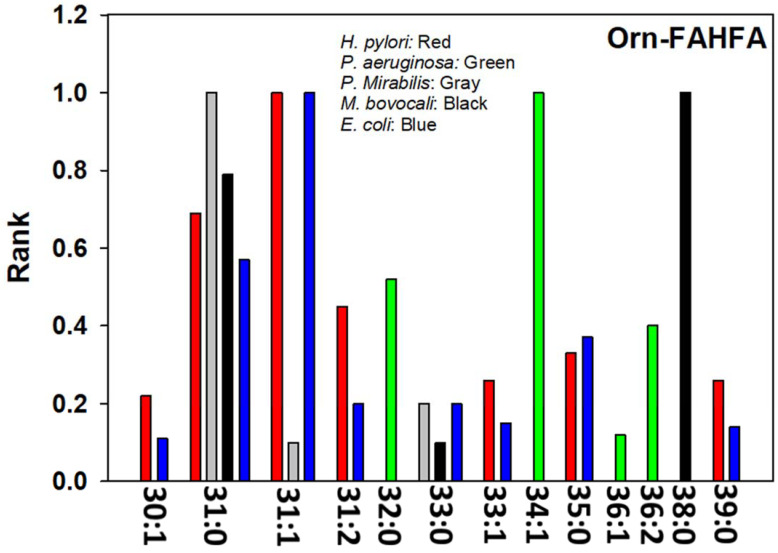
Bacterial ornithinyl-FAHFA levels presented as a rank order for each Gram-negative bacterial strain. The data are presented in Supplementary Table S1.

FAHFAs are present in mammals, but the aminoacyl forms of these lipids are not [49]. Aminoacyl FAHFAs are unique to Gram-negative bacteria. In our study, we monitored glycyl-, lysyl-, hydroxylysyl-, glutaminyl-, and ornithinyl-FAHFAs in the Gram-negative bacteria we evaluated. Orn-FAHFA (Figure 5) and Gly-FAHFA were monitored in all bacteria examined, while Ala-FAHFA was absent from *H. pylori* (Supplementary Table S2).

Gly-Ser-FAHFAs are characteristic of some Gram-negative bacteria [73,77]. We monitored these unique dipeptide lipids in *P. mirabilis* and *M. bovoculi* (Supplementary Table S2). Gly-Ser-hydroxy-fatty acids were also monitored in these two bacterial strains, as well as in *H. pylori*.

Aminoacyl FAHFAs have long been conjectured to play a role in replacing glycerophospholipids in membranes, where they regulate membrane charge. Other studies have also demonstrated their roles in signal transduction. For example, ornithine lipids act at GPCRs involved in immune activation [65]. Similarly, Gly-Ser lipids act at Toll-like 2 receptors involved in immunostimulation [69,70].

#### 3.2.2. Modified Ceramides

The addition of a polar phosphoethanolamine or phosphoglycerol group to ceramides has been shown to be another unique feature of a number of Gram-negative bacteria [64,67,73,74,75,76,77,79]. We monitored a diverse array of these lipids in *H. pylori*, *P. mirabilis*, and *M. bovocali* but not in *E. coli* or *P. aeruginosa* (Supplementary Table S2).

#### 3.2.3. Unique Sterols

Cholesteryl-acylphosphoglycosides (CPGs) have been detected in *H. pylori* [93,94] and Borella burgdorferi [95,96]. We confirm that *H. pylori* has these unique lipids and report for the first time that *P. mirabilis* also has these membrane lipids (Supplementary Table S2).

#### 3.2.4. Phosphatidyltrehalose (PT)

Phosphatidyltrehaloses have been reported for *Salmonella paratyphi* and *S. typhi* [120]. We report for the first time that these immunostimulant lipids are also present in *P. mirabilis* and *E. coli* (Supplementary Table S2).

## 4. Discussion

Our data support previous studies demonstrating the stark contrast of the lipidomes of Gram-positive and Gram-negative bacteria. Furthermore, by utilizing a standard lipid-extraction procedure, we were able to demonstrate the presence of both PDIM-B C82, a phthiodiolone dimycocerosate, and the trehalose monomycolate hTMM 28:1 in the plasma of cows with PTB. These specific constituents of the bacterial cell envelope in *M. avium* are the dominant family members we extracted from commercial bacterial pellets. Serum mycolic acids were also detected, but the levels were much more variable. Our data demonstrate the power and specificity of lipidomics to detect bacterial infections. Presumably, targeted assays to provide absolute lipid levels will provide even more specificity and sensitivity.

Lipid biomarkers have been utilized previously to demonstrate the presence of tuberculosis in archaeological samples [121,122,123,124,125] and to monitor Gram-negative bacterial infections in carotid atheroma (Gly-Ser-lipids) [73] and in oral samples from patients with periodontitis [77]. These and our current data support the idea of building a database of microbial lipids of interest to human and veterinary clinical medicine. Such a database will, in turn, yield the data required to determine which lipids might be of value to establish absolute quantitation clinical assays.

## 5. Study Limitations

This is the first step in building a comprehensive bacterial lipidomics database that will be expanded as we add the profiles of other bacteria to increase its applicability to bacterial research. Our FIA methodology has the strengths of covering a broader range of lipids and providing a stable and constant background, compared to hybrid chromatographic methods. However, issues with isobars are more prevalent with FIA. To reduce this risk, we utilized HRMS and only accepted lipids that were <1.0 ppm mass error. We also utilized MS^2^ to validate the lipid identities. The MS^2^ parameters for each lipid class are presented in Supplementary Table S3: Lincoln Memorial University, Metabolomics Unit, Flow Infusion Lipidomics Analytical Platform (Version 1.0).

## Figures and Tables

**Table 1:** Gram-positive bacterial lipoteichoic-acid-associated lipids.

Lipid Class	Bacterial Strains	References
Monohexosyl-monoacylglycerol (MHMG)	*S. mitis* and *S. oralis C. cadaveris*, *C. fallax*	[3,4,5]
Dihexosyl-MG (DHMG)	*S. pneumoniae*, *S. mitis*, *S. oralis*, *S. mutans*	[4,6]
Monohexosyl-diacylglycerol (MHDG)	*S. pneumoniae*, *S. mitis*, *S. oralis*, *C. fallax*, *S. mutans*, *Rhomboutsia* spp., *B. licheniformis*, *Clostridia* spp., *L. johnsonii*	[4,5,6,7,8,9,10]
DHDG	*S. mitis* and *S. oralis*, *C. fallax*, *S. mutans*, *Rhomboutsia* spp., *B. licheniformis*, *Clostridia* spp., *L. johnsonii*	[4,5,6,7,8,9,10]
Lipoteichoic Acid Primer (LTAP; DHDG-GroP)	*Streptococcus* spp., *B. licheniformis*, *Clostridia* spp., *Listeria* spp., *Bacillus subtilis*	[4,5,6,8,9,11]
Alanylated-LTAP (LTAP-Ala) and Di-Alanylated-LTAP	*Bacillus licheniformis*, *Bacillus subtilis*	[8,12,13]
Diglycerophosphate-DHDG (LTAdiP)	*S. pneumoniae*, *S. mitis*, *S. oralis*, *Listeria* spp.	[4,14]
Tri- and Tetra-Hexosyl-DG	*Rhomboutsia* spp., *Clostridia* spp.	[7,9]
Ala-DG	*Bacillus subtilis*, *Corynebacterium glutamicum*	[12,15,16,17]
Glucuronosyl-DG (GlcA-DG)	*Corynebacterium glutamicum*	[16,17,18]
Lysyl-DG and Lysyl-Galactosyl-DG	*Staphlococcus* spp.	[19,20]
Mannosyl-Glucuronosyl-DG (Man-GlcA-DG)	*Corynebacterium glutamicum*, *C. striatum*	[16,17,18]
N-Acetylglucosamine-DG (GlcNAc-DG)	*Clostridia* spp.	[9,19]
Phosphoethanolamine-GlcNAc-DG (PE-GlcNAc-DG)	*Clostridia* spp.	[9,21]
PE-MHDG and PE-DHDG	*Clostridia* spp.	[9,21]
Type IV LTA intermediates	Oral commensal bacteria	[4]

**Table 2:** Gram-positive bacterial aminoacyl phosphatidylglycerols (PGs).

Lipid Class	Bacterial Strains	References
Precursor CDP-DG	*Corynebacterium glutamicum*, *C. striatum*, *Clostridia* spp.	[16,17,18,22]
Lysyl-PG	*Bacillus* spp., *Clostridium* spp., *Lactobacillus* spp., *Staphylococcus* spp.	[23,24]
Alanyl-PG	*P. aeruginosa*, *Clostridia* spp., *Bifidobacteria* spp., *Staphylococci* spp., *Listeria* spp., *Bacillus* spp., *C. Corynebacterium*, *B. subtilis*	[12,13,15,16,23,24]
Leucyl-PG	*B. subtilis*	[12,13]
Succinyl-Lysyl-PG	*B. subtilis*	[12,25]
Arginyl-PG	*Enterococcus* spp., *Staphylococci* spp., *Listeria* spp., *Bacillus* spp.	[23,24]
Ornithinyl-PG	*Bacillus*, *Mycobacteria* spp	[23,24,26]
Aspartyl-PG	*B. subtilis*	[12,13]

**Table 3:** Gram-positive bacterial mycolic acids.

Lipid Class	Bacterial Strains	References
Mycolic acids (C76–C88; ᾳ-, keto-, and methoxy	*M. tuberculosis*	[29,30]
Mycolic acids (C30-46)	*R. equi*	[31]
TMM (Trehalose MonoMycolates: hydroxy, keto, acetyl)	*Corynebacterium glutamicum*, *C. striatum*, *R. equi*	[16,17,18,32]
Acyl-TMM (Mycolic-Acyl-Trehalose)	*Corynebacterium glutamicum*, *C. striatum*	[18]
TDM (Trehalose dimycolate)	*Corynebacterium glutamicum*, *C. striatum*	[18]
Acyltrehalose (MAT) and Diacyltrehalose (DAT)	*Rhodococcus ruber*, *M. tuberculosis*	[33,34]
Acyl- and Diacyl-Sulfotrehalose	*Rhodococcus ruber*, *M. tuberculosis*	[33,35,36,37]
Mycolic acid-PG (1-Mycolic-2-16:0 PG)	*Corynebacterium glutamicum*, *C. striatum*	[18]
Phthiocerol (methoxy, DIMA) / Phthiodiolone (keto, DIMB) Dimycocerosates	*Mycobacteria* spp.	[38,39]

**Table 4:** Gram-positive bacterial mannosyl phosphoinositols (PIs).

Lipid Class	Bacterial Strains	References
PIM1 (mannosyl-PI), PIM2 (dimannosyl-PI)	*Mycobacteria* spp., *Streptomyces coelicolor*, *Nocardia* spp., *Corynebacteria* spp.	[1,38,39,40,41,42]
Acyl-PIM2	*Mycobacteria*, *Corynebacteria*	[16,35,36,37,38,39,40,41]

**Table 5:** Gram-positive bacterial aminoacyl phosphatidylethanolamines (PEs).

Lipid Class	Bacterial Strains	References
Alanyl-PE	*Bacillus subtilis*	[13]
Lysyl-PE	*Bacillus subtilis*	[13]
PE Glycerol Acetals	*Clostridium fallax* but not *C. cadaveris*	[43,44,45]
GPCR Ligands	*Oral Commensal bacteria*	[5,9]

**Table 6:** Gram-negative bacterial glycosyl hydroxy fatty acids (HFAs) and fatty acyls of hydroxy fatty acids (FAHFAs).

Lipid Class	Bacterial Strains	References
Lipid A variants	*P. aeruginosa*, *E. coli*	[45,46,47,48]
Rhamnosyl- and Di-Rhamnosyl-3-HFA	*Pseudomonas* spp., *Actinetobacter calcoaceticus*, *Enterobacter asburiae*	[50,51,52,53,54]
Isopentyl metabolites	*Francisella novicida*	[55,56]
Menaquinones (MK-7, MK-8, and MK-9)	*Rhodococcus* spp., *Mycobacterium* spp., *Nocardia* spp.	[57]

**Table 7:** Gram-negative bacterial aminoacyl hydroxy fatty acids (HFAs) and fatty acyls of hydroxy fatty acids (FAHFAs).

Lipid Class	Bacterial Strains	References
Gly-FAHFA	*Bacteroidetes* spp., *Cytophaga johnsonae*	[58,59,60]
Lys-, Hydroxy-Lys-FAHFA	*Pseudobacter saltans*, *Flavobacterium johsoniae*, *Rhizobium tropici*	[61,62,63]
Orn-FAHFA	*Plantomycetes* spp., *Burkholderia* spp., *Rhizobium* spp., *Agrobacteriumtume faciens*	[1,64,65,66]
Gln-FAHFA, Gln-FAHFA(OH)	*E. coli*	[45]
Gly-Ser-FAHFA	*Flectobacillus major*, *Bacteroidetes* spp. including *P. gingivalis*	[67,68,69,70,71,72]
Gly-Ser-Orn-FAHFA, Gly-Ser-Orn-FAHFA(OH)	*Bacteroidetes* spp.	[71]
Gly-Ser-FAHFA-P-DG	*P. gingivalis*	[70,71]
Gly-Ser-HFA	*Bacteroidetes* spp., *Cryptophaga johnsonae*	[60,71,72,73]
Gly-Ser-Orn-HFA	*Bateroidetes* spp.	[71]

**Table 8:** Gram-negative-bacterial-modified ceramides.

Lipid Class	Bacterial Strains	References
Ceramide-Phosphoethanolamine (Cer-PE)	*Bacteroidetes* spp., including *P. gingivalis.* Trace levels have been monitored in mammals.	[67,74,75,76,77,78]
Cer-Phosphoinositol (Cer-PI)	*Bacteroidetes* spp.	[72,75]
Cer-Phosphoglycerol (Cer-PG)	*Bacteroidetes* spp.	[67,73,76,79]
NAPE (N-acyl-phosphatidylethanolamine)	*E. coli*, *Bdellovibrio* spp. and *Raoultella* spp.	[80,81,82,83]
Acyl-PG	*E. coli; Salmonella* spp., *Klebsiella pneumoniae*. Additionally, *C. glutamicum* Gram-positive bacteria and mammals.	[16,80,81,84,85,86,87,88,89,90,91,92,93,94,95,96,97,98,99]

**Table 9:** Gram-negative bacterial sterols.

Lipid Class	Bacterial Strains	References
Cholesteryl Acyl ᾳ-Glycoside (CAG)	*Helicobacter pylori*, *Borrelia burgdorferi*	[93,94,95,96]
Cholesteryl Acyl ᾳ-Phospho-Glycoside (CPG)	*Helicobacter pylori*, *Borrelia burgdorferi*	[93,94,95,96]
Cholesteryl Phosphoethanolamine-Glycoside (CEPG)	*Helicobacter pylori*	[94]
Bacteriohopanetetrol cyclitol ethers (BHT-CE)	*Burkholderia* spp., *Methylobacterium organophilum*, *Chloracidobacteria* spp.	[98,99,100,101]

**Table 10:** Gram-Negative Bacterial Secondary Metabolites.

Lipid Class	Bacterial Strains	References
Undecylprodigiosin metabolites	*Streptomyces* spp., *Serratia marcescens*	[102,103,104]
Malleilactone	*Burkholderia pseudomallei*	[105]

## Data Availability

All data are in the manuscript.

## Supplementary Materials

The following supporting information, in a single file, can be downloaded at https://www.mdpi.com/article/10.3390/metabo13070809/s1. Table S1: Rank order of lipid families in Gram-positive bacteria. Table S2: Rank order of lipid families in Gram-negative bacteria. Table S3: Lincoln Memorial University, Metabolomics Unit, Flow Infusion Lipidomics Analytical Platform (Version 1.0).
