# Supplementary material for: Construction of a Bacterial Lipidomics Analytical Platform: Pilot Validation with Bovine Paratuberculosis Serum
Source: Metabolites. 2023 Jun 29;13(7):809. doi: 10.3390/metabo13070809 (PMC10383236; doi:10.3390/metabo13070809)
Supplement: Supplementary file 1 [file metabolites-13-00809-s001.zip › metabolites-2428143-supplementary.pdf]

Supplementary Table S1. Rank order of lipid families in Gram-positive bacteria. For the most abundant family member (i.e. 1.0), the number in brackets is the ratio of the peak area of the lipid to the peak area of 2 nanomoles of [$^{13}C_3$]Diacylglycerol 36:2.

Lipid	ESI	*M. avium (PTB)*	*S. aureus*	*M. bovis*	*M. smegmatis*	*R. equi*	*E. faecalis*	*C. glutamicum*
**ATTC #**		700535	10832	35737	14468	7699	19433	13032
Class		*Actinomyceta*	*Bacilli*	*Actinomyceta*	*Actinomyceta*	*Actinomyceta*	*Bacilli*	*Actinomyceta*
Metabolism		Aerobe	Anaerobe	Aerobe	Aerobe	Aerobe	Anaerobe	Aerobe
**Phthiodiolone Dimycocerates**	**[M+Cl]⁻**	***M. avium (PTB)***	***S. aureus***	***M. bovis***	***M. smegmatis***	***R. equi***	***E. faecalis***	***C. glutamicum***
PDIM-B C82	1260.1964	1.0 (0.65)						
PDIM-B C83	1274.2121	0.062						
PDIM-B C84	1288.2277	0.39						
**Diacyltrehaloses (Mycolipanolic)**	**[M+Cl]⁻**	***M. avium (PTB)***	***S. aureus***	***M. bovis***	***M. smegmatis***	***R. equi***	***E. faecalis***	***C. glutamicum***
DAT2 C51	967.6498	0.67						
DAT2 C52	981.6655	1.0 (2.3)						
DAT2 C53	995.6811	0.020						
DAT2 C54	1009.6968	0.032						
DAT2 C58	1065.7594	0.015						
**Trehalose -OH-Monomycolates**	**[M+Cl]⁻**	***M. avium (PTB)***	***S. aureus***	***M. bovis***	***M. smegmatis***	***R. equi***	***E. faecalis***	***C. glutamicum***
hTMM 24:0	743.4358	0.033						
hTMM 24:1	741.4202					1.0 (0.98)		
hTMM 26:0	771.4671	0.37						
hTMM 28:1	797.4828	1.0 (0.89)						
hTMM 28:2	795.4671	0.013						
hTMM 29:1	811.4984							0.0094
hTMM 34:1	881.5767			1.0 (4.8)				0.0074
hTMM 34:2	879.5610			0.10				0.048
hTMM 35:0	897.6080			0.70				
hTMM 38:0	939.6549			0.002				1.0 (0.47)
hTMM 38:2	935.6236					0.014		

Trehalose Monomycolates (Acetyl)	[M+Cl]-	*M. avium (PTB)*	*S. aureus*	*M. bovis*	*M. smegmatis*	*R. equi*	*E. faecalis*	*C. glutamicum*
Ac-TMM 26:0	813.4777							0.026
Ac-TMM 27:3	821.4464							0.43
Ac-TMM 28:0	841.5090	0.15						
Ac-TMM 31:0	883.5559	0.012						
Ac-TMM 32:0	897.5716	0.052			1.0 (0.013)			
Ac-TMM 32:1	895.5559			0.16				
Ac-TMM 33:0	911.5872	0.22		1.0 (0.15)				
Ac-TMM 33:2	907.5559							0.10
Ac-TMM 34:0	925.6029	0.030						
Ac-TMM 35:0	939.6185	0.58						
Ac-TMM 35:1	937.6029	0.025						
Ac-TMM 36:0	953.6342	1.0 (1.97)						
Ac-TMM 37:0	967.6498	0.28						
Ac-TMM 37:1	965.6342							1.0 (0.039)
Ac-TMM 37:2	963.6185							0.064
Ac-TMM 38:0	981.6655	0.42						
**Lipoteichoic Acid Precursor**	**[M-H]-**	***M. avium (PTB)***	***S. Aureus***	***M. bovis***	***M. smegmatis***	***R. equi***	***E. faecalis***	***C. glutamicum***
LTAP 28:1	987.5299					1.0 (0.058)		
LTAP 31:0	1031.5925					0.27		
LTAP 32:0	1045.6082		1.0 (0.004)					
LTAP 32:1	1043.5925						0.29	
LTAP 34:1	1071.6238						1.0 (0.012)	
LTAP 34:2	1069.6082						0.18	
LTAP 35:2	1083.6238						0.15	
LTAP 36:0	1115.6864							0.20
LTAP 36:3	1109.6395							1.0 (0.0050)
LTAP 38:4	1135.6551					0.37		
**Alanyl-LTAP**	**[M-H]-**	***M. avium (PTB)***	***S. aureus***	***M. bovis***	***M. smegmatis***	***R. equi***	***E. faecalis***	***C. glutamicum***

Ala-LTAP 31:0	1102.6296						0.49	1.0 (0.18)
Ala-LTAP 33:0	1130.6609					1.0 (0.11)	1.0 (0.21)	
Ala-LTAP 33:1	1128.6453					0.66		
Ala-LTAP 33:2	1126.6296					0.023		
Ala-LTAP 34:0	1144.6766							0.45
**Di-Alanyl-LTAP**	**$[M-H]^-$**	***M. avium (PTB)***	***S. aureus***	***M. bovis***	***M. smegmatis***	***R. equi***	***E. faecalis***	***C. glutamicum***
Di-Ala-LTAP 36:0	1243.7450					0.31		
Di-Ala-LTAP 38:0	1271.7763					1.0 (0.045)		
**Acyl-PIM1**	**$[M-H]^-$**	***M. avium (PTB)***	***S. aureus***	***M. bovis***	***M. smegmatis***	***R. equi***	***E. faecalis***	***C. glutamicum***
Ac-PIM1 44:3	1147.6915							0.49
Ac-PIM1 44:4	1145.6758							0.95
Ac-PIM1 45:3	1161.7071							0.43
Ac-PIM1 45:4	1159.6915							1.0 (0.23)
Ac-PIM1 46:3	1175.7228							0.38
Ac-PIM1 46:4	1173.7071							0.95
Ac-PIM1 47:4	1187.7228							0.76
Ac-PIM1 48:4	1201.7384							0.42
Ac-PIM1 49:4	1215.7541							0.51
**Mycolic Acids**	**$[M+Cl]^-$**	***M. avium (PTB)***	***S. aureus***	***M. bovis***	***M. smegmatis***	***R. equi***	***E. faecalis***	***C. glutamicum***
M 24:0	419.3302	0.32						1.0 (0.044)
M 24:1	417.3145							
M 26:0	447.3615	0.26		0.31				
M 26:2	443. 3302		1.0 (0.084)				1.0 (0.11)	
M26:3	441.3145						0.12	
M 28:0	475.3928	0.48						
M 28:1	473.3771							0.10
M 28:2	471.3615		0.12					
M 30:0	503.4241	0.45						
M 32:0	531.4554	1.0 (0.19)						0.83
M 32:1	529.4397	0.051						

M 34:0	559.4867	0.20						
M 34:1	557.4710	0.029						0.36
M 34:2	555.4554		0.12					
M 35:1	571.4867		0.14					
M 42:2	667.5806			0.14				
M 44:2	695.6119			0..26				
M 46:1	725.6588			0.30		1.0 (0.086)		
M 46:2	723.6432			0.54				
M 48:1	753.6901			0.21				
M 48:2	751.6745			0.52				
M 50:2	779.7058			0.60		0.13		
M 52:1	809.7527			0.16				
M 52:2	807.7371			0.37				
M 54:2	835.7684			0.19				
M 58:2	891.8310			0.37				
M 59:2	905.8466			0.21				
M 60:1	886.9081				0.024			
M 60:2	919.8623			0.46				
M 61:2	933.8779			0.10				
M 62:2	947.9092				0.27			
M 64:2	975.9249				1.0 (0.37)			
M 66:3	1001.9405				0.23			
M 68:2	1031.9875				0.13			
M 68:3	1029.9718				0.28			
M 70:2	1060.0188				0.043			
M 70:3	1058.0031				0.66			
M 72:2	1088.0501				0.25			
M 72:3	1086.0344				0.093			
M 73:0	1106.0970					0.012		
M 73:2	1102.0657				0.072			
M 74:2	1116.0814				0.31			
M 74:3	1114.0657				0.37			
M 75:2	1130.0970				0.28			

M 75:3	1128.0814				0.080			
M 76:2	1144.1127			0.35	0.26			
M 76:3	1142.0970				0.49			
M 77:1	1125.1742			0.13				
M 77:2	1123.1585			0.17				
M 77:3	1156.1127				0.25			
M 78:2	1172.1440			1.0 (0.058)				
M 78:3	1170.1283				0.33			
M 79:2	1186.1596			0.17				
M 79:3	1184.1440				0.17			
M 80:2	1200.1753	0.078						
M 80:3	1198.1586				0.069			
M 82:2	1228.2066	0.034						
**Epoxy / Keto / Methoxy(cyclopropyl) Mycolic Acids**	**$[M-H]^-$**	***M. avium (PTB)***	***S. aureus***	***M. bovis***	***M. smegmatis***	***R. equi***	***E. faecalis***	***C. glutamicum***
E-K-MC 77:1	1174.1233				1.0 (0.034)			
E-K-MC 78:1	1188.1389				0.37			
E-K-MC 80:0	1218.1859			0.21				
E-K-MC 82:0	1246.2172			1.0 (0.022)				
**Dicarboxylic Mycolic Acids**	**$[M-H]^-$**	***M. avium (PTB)***	***S. aureus***	***M. bovis***	***M. smegmatis***	***R. equi***	***E. faecalis***	***C. glutamicum***
DC-Mycolic 64:2	969.9220			0.62				
DC-Mycolic 82:1	1224.2193	1.0 (0.164)						
DC-Mycolic 84:1	1252.2506	0.12						
DC-Mycolic 86:2	1278.2663			0.31				
DC-Mycolic 86:3	1276.2506			1.0 (0.016)				
DC-Mycolic 88:0	1310.3289						1.0 (0.12)	

**Sulfonolipids**	**$[M-H]^-$**	***M. avium (PTB)***	***S. aureus***	***M. bovis***	***M. smegmatis***	***R. equi***	***E. faecalis***	***C. glutamicum***
Sulfonolipid 32:0;O	574.4511			0.529				0.356

Sulfonolipid 32:1;O	572.4354			0.052				
Sulfonolipid 33:1;O2	602.4460			0.081				
Sulfonolipid 33:2;O2	600.4303			0.027				1.0 (0.11)
Sulfonolipid 34:1;O2	616.4616			0.498				0.367
Sulfonolipid 34:0;O2	618.4773			1. 0 (0.040)				0.630
Sulfonolipid 35:1;O2	630.4773			0.732				
Sulfonolipid 37:2;O2	656.4929							0.025
**AAHFA**	**[M-H]-**	***M. avium* *(PTB)***	***S. aureus***	***M. bovis***	***M. smegmatis***	***R. equi***	***E. faecalis***	***C. glutamicum***
AAHFA 25:0	411.3480	0.024	0.017	0.452				
AAHFA 26:0	425.3636	1.0 (1.73)	0.068	0.219				
AAHFA 27:0	439.3793	0.022		1.0 (0.079)				
AAHFA 28:0	453.3949	0.005	1.0 (0.13)	0.023				
AAHFA 29:0	467.4106		0.393					
AAHFA 30:0	481.4262	0.042	0.130	0.009				
AAHFA 31:0	495.4419		0.769					
**Glcopeptidolipids**	**[M+Cl]-**	***M. avium* *(PTB)***	***S. aureus***	***M. bovis***	***M. smegmatis***	***R. equi***	***E. faecalis***	***C. glutamicum***
h26:1(DiAc-dTal)-Phe-Thr-Ala-Alaninol-TriMe-Rham	1239.7408							0.134
h28:0(Ac-dTal)-Phe-Thr-Ala-Alaninol-DiMe-Rham	1213.7615							1.0 (0.48)
h28:1(DiAc-dTal)-Phe-Thr-Ala-Alaninol-Me-Rham	1239.7408							0.134
h28:1(DiAc-dTal)-Phe-Thr-Ala-Alaninol-DiMe-Rham	1253.7564							0.122
h30:1(Ac-dTal)-Phe-Thr-Ala-Alaninol-DiMe-Rham	1239.7772				0.0079			

h30:1(DiAc-dTal)-Phe-Thr-Ala-Alaninol-DiMe-Rham	1281.7877					0.00435		0.111
h30:1(DiAc-dTal)-Phe-Thr-Ala-Alaninol-TriMe-Rham	1295.8034				0.0627			
h30:1(Ac-dTal)-Phe-Thr-Ala-Alaninol-TriMe-Rham	1253.7928				0.0739	1.0 (0.22)		
h32:1(dTal)-Phe-Thr-Ala-Alaninol-Me-Rham	1211.7823			1.0 (0.0019)				
h32:1(Ac-dTal)-Phe-Thr-Ala-Alaninol-DiMe-Rham	1267.8085				0.6321			
h32:1(DiAc-dTal)-Phe-Thr-Ala-Alaninol-Rham	1281.7877			0.0024		0.00435		
h32:1(DiAc-dTal)-Phe-Thr-Ala-Alaninol-Me-Rham	1295.8034				0.0627			
h32:1(Ac-dTal)-Phe-Thr-Ala-Alaninol-TriMe-Rham	1281.8241			0.3796	1.0 (0.25)	0.39964		
h34:1(Ac-dTal)-Phe-Thr-Ala-Alaninol-DiMe-Rham	1295.8398				0.7036	0.17345		
h34:1(Ac-dTal)-Phe-Thr-Ala-Alaninol-TriMe-Rham	1309.8554				0.9959			

Supplementary Table S2. Rank order of lipid families in Gram-negative bacteria. For the most abundant family member (i.e. 1.0), the number in brackets is the ratio of the peak area of the lipid to the peak area of 2 nanomoles of [$^{13}C_3$]Diacylglycerol 36:2.

**Lipid**		***H. pylori***	***P. aeruginosa***	***P. Mirabilis***	***M. bovoculi***	***E. coli***
**ATCC #**		***43504***	**10145**	**12453**	**19965**	**12435**
Class		Camylobacteria	γ-proteobacteria	γ-proteobacteria	γ-proteobacteria	γ-proteobacteria
Metabolism		Aerobe	*Aerobe*	Anaerobe	Anaerobe	*Anaerobe*
**Ornithinyl-FAHFA**	**$[M+H]^+$**					
ORN-FAHFA 30:1	595.5045	0.22				0.11
ORN-FAHFA 31:0	611.5358	0.69		1.0 (0.20)	0.79	0.57
ORN-FAHFA 31:1	609.5201	1.0 (0.15)		0.055		1.0 (0.24)
ORN-FAHFA 31:2	607.5045	0.45				0.20
ORN-FAHFA 32:0	625.5514		0.52			
ORN-FAHFA 33:0	639.5671		0.052	0.23	0.070	0.20
ORN-FAHFA 33:1	637.5514	0.26				0.15
ORN-FAHFA 34:1	651.5671		1.0 (5.19)			
ORN-FAHFA 35:0	667.5984	0.33				0.37
ORN-FAHFA 36:1	679.5984		0.12			
ORN-FAHFA 36:2	677.5827		0.40			
ORN-FAHFA 38:0	709.6453				1.0 (0.36)	
ORN-FAHFA 39:0	723.6297	0.26				0.14
**Glycyl FAHFA**	**$[M+H]^+$**	***H. pylori***	***P. aeruginosa***	***P. Mirabilis***	***M. bovoculi***	***E. coli***
Gly-FAHFA 30:1	538.4466	0.015				
Gly-FAHFA 32:0	568.4936				1.0 (0.21)	
Gly-FAHFA 33:1	580.4936	0.11				
Gly -FAHFA 34:0	596.5249	1.0 (.21)				
Gly -FAHFA 34:1	594.5092	0.25				1.0 (0.024)
Gly-FAHFA 36:0	610.5405	0.21	1.0 (0.069)	1.0 (0.54)	0.29	0.47
Gly-FAHFA 36:1	608.5249				0.22	
**Lysyl-FAHFA**	**$[M-H]^-$**	***H. pylori***	***P. aeruginosa***	***P. Mirabilis***	***M. bovoculi***	***E. coli***
Lys-FAHFA 31:0	623.5369		0.61			
Lys-FAHFA 33:1	649.5525		1.0 (0.58)			
Lys-FAHFA 34:1	663.5682		0.62			

Lys-FAHFA 36:2	675.5682		0.34			
**Hydroxy-Lysyl-FAHFA**	**[M-H]-**	***H. pylori***	***P. aeruginosa***	***P. Mirabilis***	***M. bovoculi***	***E. coli***
OH-Lys-FAHFA 31:2	635.5005				0.066	
OH-Lys-FAHFA 32:1	651.5318			0.035		
OH-Lys-FAHFA 33:1	665.5474			0.89		
OH-Lys-FAHFA 34:0	681.5787				1.0 (0.017	
OH-Lys-FAHFA 34:1	679.5631			1.0 (0.011)		
**Gycine-Serine-Hydroxy-FA**	**[M+H]+**	***H. pylori***	***P. aeruginosa***	***P. Mirabilis***	***M. bovoculi***	***E. coli***
Gly-Ser-HFA 16:0	431.3116			0.41	0.025	
Gly-Ser-HFA 17:0	445.3272	0.20		1.0 (0.013)	1.0 (0.19)	
Gly-Ser-HFA 18:0	459.3429	1.0 (0.081)		0.19	0.59	
**Glycine-Serine-FAHFA**	**[M-H]-**	***H. pylori***	***P. aeruginosa***	***P. Mirabilis***	***M. bovoculi***	***E. coli***
Gly-Ser-FAHFA 30:0	625.4797			0.21	0.014	
Gly-Ser-FAHFA 31:0	639.4954			0.011	0.19	
Gly-Ser-FAHFA 32:0	653.5110				1.0 (0.057)	
Gly-Ser-FAHFA 33:0	667.5267			1.0 (0.073)		
Gly-Ser-FAHFA 34:0	681.5423			0.41		
**Glutaminyl-FAHFA**	**[M-H]-**	***H. pylori***	***P. aeruginosa***	***P. Mirabilis***	***M. bovoculi***	***E. coli***
Gln-FAHFA 31:0	623.5005				0.28	
Gln-FAHFA 32:1	635.5005				1.0 (0.0011)	
Gln-FAHFA 34:0	665.5474			0.89		
Gln-FAHFA 36:0	679.5631			1.0 (0.011)		
**Rhammnosyl-FAHFA**	**[M-H]-**	***H. pylori***	***P. aeruginosa***	***P. Mirabilis***	***M. bovoculi***	***E. coli***
Rham-FAHFA 20:0	503.3226		1.0 (0.52)			
Rham-FAHFA 21:0	517.3382		0.055			
diRham-FAHFA 18:0	621.3492			1.0 (0.010)		
diRham-FAHFA 22:0	677.4118		0.023			
**Phytoceramide-PE**	**[M-H]-**	***H. pylori***	***P. aeruginosa***	***P. Mirabilis***	***M. bovoculi***	***E. coli***
PE-Cer 34:5;O3	667.4457			0.061		
PE-Cer 35:2;O3	687.5083	1.0 (0.08)				
PE-Cer 36:3;O3	699.5083	0.010				
PE-Cer 36:5;O3	695.4770			1.0 (0.022)		
PE-Cer 40:2;O3	757.5865			0.11		

**Phytoceramide-PG**	**[M-H]⁻**	***H. pylori***	***P. aeruginosa***	***P. Mirabilis***	***M. bovoculi***	***E. coli***
PG-Cer 32:1;O3	678.4715			0.018		
PG-Cer 32:3;O3	674.4402	0.90				
PG-Cer 35:1;O3	720.5185				0.53	
PG-Cer 36:2;O3	732.5185			0.17		
PG-Cer 38:3;O3	758.5341	1.0 (0.0042)				
PG-Cer 40:2;O3	786.5654			0.087		
PG-Cer 42:2;O3	814.5967			0.058		
PG-Cer 44:2;O3	842.6280	0.16				
PG-Cer 44:3;O3	840.6124				0.17	
PG-Cer 46:3;O3	868.6437				1.0 (0.0085)	
PG-Cer 46:4;O3	866.6280	0.21				
PG-Cer 48:1;O3	900.7063			0.072		
PG-Cer 48:2;O3	898.6906			0.15		
PG-Cer 50:2;O3	926.7219			1.0 (0.061)		
**Ceramide-PG**	**[M-H]⁻**	***H. pylori***	***P. aeruginosa***	***P. Mirabilis***	***M. bovoculi***	***E. coli***
PG-Cer 32:2;O2	660.4610	1.0 (0.15)				
PG-Cer 32:3;O2	658.4453				0.36	
PG-Cer 34:1;O2	690.5079			0.26		
PG-Cer 34:3;O2	686.4766	0.23				
PG-Cer 36:2;O2	716.5236			1.0 (0.83)	0.83	
PG-Cer 38:3;O2	742.5392			0.12	1.0 (0.37)	
PG-Cer 38:4;O2	740.5236				0.059	
PG-Cer 46:1;O2	856.6801	0.16				
**Cholesteryl-Ptd-Glucosides**	**[M-H]⁻**	***H. pylori***	***P. aeruginosa***	***P. Mirabilis***	***M. bovoculi***	***E. coli***
CPG 31:5	1151.7533	0.66				
CPG 32:5	1165.7690	1.0 (1.67)				
CPG 33:2	1185.8316	0.015				
CPG 33:5	1179.7846	0.028		0.094		
CPG 34:5	1193.8003	0.016				
CPG 34:6	1191.7846			0.028		
CPG 36:6	1219.8159	0.022				
CPG 38:4	1251.8785			0.15		

CPG 40:4	1279.9098			1.0 (0.0053)		
CPG 40:5	1277.8942			0.25		
CPG 40:6	1275.8785			0.021		
**Ptd-Trehalose**	**[M+Cl]$^-$**	***H. pylori***	***P. aeruginosa***	***P. Mirabilis***	***M. bovoculi***	***E. coli***
Ptd-Trehalose 38:0	1091.6424			1.0 (0.17)		1.0 (1.29)
Ptd-Trehalose 40:0	1119.6737			0.019		
**Ceramide Sulfates**	**[M-H]$^-$**	***H. pylori***	***P. aeruginosa***	***P. mirabilis***	***M. bovoculi***	***E. coli***
Cer Sulfate 32:0;O (Sulfobactin B)	574.4511			0.030		
Cer Sulfate 32:1;O	572.4354					
Cer Sulfate 32:1;O2	588.4303					
Cer Sulfate 33:1;O2	602.4460					
Cer Sulfate 33:2;O2	600.4303			0.143		
Cer Sulfate 35:0;O	616.4980	0.012				
Cer Sulfate 34:1;O2 (Flavocristamide A)	616.4616					
Cer Sulfate 34:0;O2 (Sulfobacin-A)	618.4773			1.0 (0.023)		
Cer Sulfate 35:1;O2	630.4773	1.0 (1.85)		0.045		
Cer Sulfate 35:2;O2	628.4616					
Cer Sulfate 36:1;O2	644.4929			0.024		
Cer Sulfate 37:2;O2	656.4929			0.030		
**AAHFA**	**[M-H]$^-$**	***H. pylori***	***P. aeruginosa***	***P. mirabilis***	***M. bovoculi***	***E. coli***
AAHFA 25:0	411.3480			0.615		
AAHFA 26:0	425.3636			1.0 (0.0091)		1.0 (0.34)
AAHFA 27:0	439.3793			0.046		
AAHFA 28:0	453.3949			0.162		
AAHFA 29:0	467.4106					
AAHFA 30:0	481.4262			0.016		
AAHFA 31:0	495.4419			0.615		

Table S3. LMU Metabolomics Unit Flow Infusion Lipidomics Analytical Platform (Version 1.0)

Scans:

- **Infusion solvent (**2-propanol:methanol:chloroform (8:4:4), containing 5 mM ammonium chloride)
- **HR-MS (140,000)**
- **NESI: 290 – 1500**
- **PESI: 290 – 1500**
- **Lipids with < 1 ppm mass error reported**

Sources: LipidMaps [PMID 33037133], E. coli Metabolome Database (ECMDB) [PMID 26481353], Yeast Metabolome Database (YMDB) [PMID 27899612], Mycobacterium tuberculosis Database (Mtb LipidDB) [PMID 21285232], Chemical Entities of Biological Interest (ChEBI) [PMID 26467479], Human Metabolome Database (HMDB) [PMID 34986597], Seaweed Metabolite Database (SWMD) [PMID 21423723], PubChem [PMID 33151290], PubMed [PMID 33085945], original publications, and our experience with commercial analytical standards and bacterial extracts.

Shortforms:

Ac, acetyl; Cer, ceramide; e, dTal, deoxytalose; ether (alkyl); FA, fatty acid; FAHFA, fatty acyl of hydroxy fatty acid; GroP, glycerol phosphate; h, hydroxy; HFA, hydroxy-fatty acid; Me, methyl; p, plasmalogen (alkenyl); PC, phosphatidylcholine; PE, phosphatidyl ethanolamine, Pep, Phe-Thr-Ala-Alaninol; PI, phosphatidyl inositol; PG, phosphatidylglycerol; PS, phosphatidul serine; Rham, rhamnose;

Isobar Issues with Flow Infusion:

Most of the listed issues require chromatography and/or MS/MS for resolution. In some cases, the ionization can resolve the issue. For example, with the SM 41:2;O2 = Cer-PE 44:1;O2 case these lipids have identical $[M+H]^+$ ions while in NESI, the SM forms a chloride adduct while the Cer-PE has a $[M-H]^-$ ion. Similarly, while PC and PE form $[M+H]^+$ ions in PESI, in NESI PE forms a $[M-H]^-$ ion while PC forms a chloride adduct.

- Cyclopropyl group in an FA chain is equivalent to a double bond (i.e. loss of 2H).
- Oxygenated fatty acid issues: keto = epoxy = methoxy + a double bond
- Branched chain FA is not distinguished from a straight chain FA.
- Galactosyl-, glucosyl-, mannosyl-, & inositol-substitutions cannot be distinguished.
- A plasmalogen is equivalent to an ether lipid with an unsaturated FA substitution.
- PE xx:1 = NAPEp xx:0 = PE xx-3:1
- Diacyl-GPG: Diacyl substitution on 1 PG = a single acyl substitution on each of the 2 PGs.

- Cer-PG;O3 + PSp & Cer-PG;O2 = PE
- DG-AEP = PEe
- SM 41:2;O2 = Cer-PE 44:1;O2
- ketoTMM xx:0 = hTMM xx:1 (h = hydroxy)
- NAE 16:0 = sphingosine = ketosphingosine
- Hex-Cer xx:1 = DGCC xx-4:0
- PI xx:0 = Gal-GroP xx:0
- Cer d18:1:xx = d18:0:xx+1
- DG xx = TGalkyl-diacyl (DAGE) xx
- Amino acylation issues:
  - Trimethylhomoserine = hydroxymethyl-trimethylalanine
  - Leu-PG xx:3 = Amadori-PEp xx:4.
  - Gly-Ser-FAHFA = Gln-FAHFA(OH)
  - Asp-PG xx:0 = PE-MHDG xx-1:1
  - Hydroxy-Lys-Lipid = Lys-Hydroxylipid
  - Gly-Ser-Lipid = Gln-Hydroxylipid
  - MGTS = MGTA

Key Reviews & Methods Publications:

- **LipidMaps nomenclature:** PMID 15722563, 33037133
- **Mass Spectrometry :** PMID 27760388, 35416821
- **Bacterial Lipid Diversity:** PMID 25862689, 28510205, 29446847
- **Lipoteichoic Acids:** PMID 27867802,
- **Mycobacterial Lipids:** PMID 34929571,
- ***C. glutamicum* Lipids:** PMID 2972478, 33954950
- **Betaine Lipids:** PMID 19703488

Request:

A real issue with mega data is that a significant number of errors end up in published literature. This analytical platform is continuously updated based on experience with both analytical standards and biological samples. The authors request that readers report to us (paul.wood@lmunet.edu) any errors they find in the platform. Refinements and updates also would be appreciated.

Fatty Acyls (FA)

- Unsaturated fatty acids [FA0102]:
  - **[M-H]⁻**
- Heterofibrins [FA0103]:
  - **[M-H]⁻ → [Lactic acid]⁻ = [89.0244]⁻ , [Propionic acid]⁻ = [73.0294]⁻ & cleavage at the diyne-ene moiety closest to the carboxy terminal resulting in product ions of [173.1336]⁻ for HF-A2 and [187.1492]⁻ for HFB2**
  - Lipid drop inhibitors isolated from sponges [PMID 20526092, 21857959] & copepods [PMID]
  - Fatty acids with diyne-ene moiety & monolactyl or dilactyl esters
- HFA (Hydroxy fatty acids) [FA0105]:
  - **[M-H]⁻**
- VLCFA (Very-Long-Chain FA):
  - **[M+Cl]⁻ → [M]⁻**
  - **[M+NH4]⁺ → [M + H]⁺**
- Mycolic acids (MA) [FA0116]:
  - Hydroxy, epoxy, methoxy, & keto species with o, 1, or 2 cyclopropane rings
  - Accurate masses [PMID 11575804]
  - **[[M+Cl]⁻ → [M-H]⁻ & [FA]⁻**
  - **[M+NH4]⁺ → [MH-H_2O & - 2H_2O]⁺ & [MH-FA]⁺**
  - Gram-positive *Mycobacteria spp.* [PMID 21285232, 24374164, 26319139, 26574042, 31026154, 34929571], *R. equi* [PMID 20946862], *C. glutamicum* [PMID 22538651], and *S. typhi* [PMID 34600165]
  - Major lipids of both outer & inner leaflets & cell envelope [PMID 28327797]
  - Free mycolic acids are present in mycobacterial biofilms [PMID 29403446]
  - Mycolipenic acids [FA0102]:
    - Analysis after base hydrolysis of archaeological samples [PMID 22860031, 25797611, 33338873]
  - Mycolipanolic acid substituents
    - Isobars: Hydroxy fatty acids & hydroxy mycocerosic acids
    - Isobar Resolution: Chromatography
  - Mycocerosic acids
    - Isobars: Phthioceranic acids

    - Isobar Resolution: Chromatography
- MA-PG (1-Mycolic Acid-2-Acyl-Phosphatidylglycerol):
    - **$[M-H]^-$ → $[Mycolic\ acid]^-$ & $[FA]^-$**
    - Gram-positive C. glutamicum & C. striatum [PMID 32659445, 33954950]
- PDIM (Phthierol = PDIM-A (-O-CH_3)/Phthiodiolone = PDIM-B (=O) Dimycocerosate Esters):
    - Long chain dihydroxy glycol (phthiocerol) diesterified with long-chain mycoceroisic/mycoceranic acids
    - **$[M+NH4]^+$ → $[MH - FA]^+$ & $[MH - FA – methoxy]^+$ = $[MH -32.0262]^+$**
    - *Mycobacteria* [PMID 26574042, 33629944]
- Trehalose lipids
    - Phosphatidyl trehalose
        - **$[M+NH_4]^+$ → $[FA]^+$ & $[MH - hexose]^+$ & $[MH – trehalose]^+$**
        - *Salmonella* Gram-negative bacteria [PMID30804000]
    - TMM (Trehalose mycolates): hydroxy, keto, & methoxy
        - **$[M+Cl]^-$**
        - **$[M+NH4]^+$ → $[MH- hexose]^+$ & $[Mycolic\ acid\ \&\ mycolic\ acid\ - H_2O]^+$**
        - Isobars: e.g. KetoTMM 24:0 = hTMM 24:1
        - Isobar Resolution: MS^2
        - *Rhodococcus equi* [PMID 21972013] & *Mycobacteria spp.* Gram-positive bacteria [PMID 12753183, 15870454, 21972013, 29724782, 33954950,34600165]
    - hGMM (Glucosyl-MonoHydroxycorynomycolate):
        - **$[M+NH4]^+$ →**
    - MAT (Monoacyl Trehalose):
        - **$[M+Cl]^-$ → $[M - FA]^-$, $[FA]^-$ & $[Trehalose]^-$ = $[341.1089]^-$**
        - **$[M+NH4]^+$ → $[MH - hexose]^+$ & $[FA - H_2O]^+$**
        - *Rhodococcus ruber* Gram-positive bacteria; suppress antibody & cytokine production [PMID 32910375]
    - DAT (Diacyltrehalose) [SL03]:
        - **$[M+Cl]^-$ → $[M - FA]^-$, $[FA]^-$ & $[Trehalose]^-$ = $[341.1089]^-$**
        - **$[M+NH4]^+$ → $[MH - hexose]^+$ & $[M - FA]^+$**
        - *M. tuberculosis* [PMID 33491458]
    - Trehalose Sulfoglycolipids (SGL) [SL03]:
        - Acyl-Sulfotrehalose (AcSGL):

        - [M-H]$^-$
        - Mycobacteria [PMID 175921143]
    - Ac2SGL (Diacyl-Sulfotrehalose):
        - [M-H]$^-$
        - Mycobacteria [PMID 14981115, 21482713, 22194604]
- Phosphatidyltrehalose (Trehalose-P-DG):
    - **[M-H]$^-$ → [FA from the HFA]$^-$, [PA]$^-$, [M – hexose]$^-$ = [M - 180.0634]$^-$ & [M – (Tehalose – H_2O)]$^-$= [M -324.1056]$^-$**
    - *Mycobacteria* Gram-positive bacteria & *Salmonella spp.* Gram-negative bacteria [PMID 30804000]. S. typhi
    - Immunostimulant
- MT-MM (Maltotriosmonomycolate)
    - **[M+Cl]$^-$**
    - *C. glutamicum Gram positive bacteria*

➢ DCA (Dicarboxylic acids)[FA0117]:

- **[M-H]$^-$ → [M-H - H_2O]$^-$ & [M-H - H_2O - CO_2]$^-$**
- VLCDCA are anti-inflammatory & anti-proliferative lipids [PMID30400281]

➢ Fatty alcohols [FA05]

- Bacilli wax diols:
    - **[M-H]$^-$**
    - Phthiocerols
    - Phthiotriols
    - Phthodiones

➢ N-Acyl Amino Acids

- N-3-hydroxypalmitoyl glycine (Commendamide): GPR132 ligand
- **[M-H]$^-$ → [Gly]$^-$ = [74.0248]$^-$ & [Gly-CO-CH_2]$^-$ = [116.0353]$^-$**
- N-3-hydroxypalmitoyl ornithine: SIPR4 ligand
- **[M-H]$^-$ → [Orn]$^-$ = [131.0826]$^-$**
- Acyl-Serinols [FA0902]:
    - **[M+Cl]$^-$**
    - **[M+H]$^+$ → [Serinol]$^+$ = [92.0706]$^+$ & [MH – (FA – H_2O)]$^+$**
    - Produced by skin Gram-positive commensal bacteria [PMID 28854168]
    - Act as endocannabinoids to augment ceramide levels & tighten epidermal tight junctions [PMID 28854168, 34248264, 10617602]

        - Oleoyl serinol acts at GPR119 receptor [PMID 28854168]
    - NAE (N-Acylethanolamides):
        - **$[M+H]^+$ → $[Ethanolamine]^+$ = $[62.0600]^+$**
    - Siolipins:
        - **$[M+H]^+$**
        - CID 139584800, 42607284
        - Gram-positive Stretomyces sioyaensis [PMID 5784124]
- Wax Esters: **$[M+NH4]^+$**
    - Wax monoesters [FA0701]
        - **$[M+NH4]^+$ → $[FA\ \&\ FA - H_2O]^+$**
        - Mass spectrometry [PMID 17277382, 26178197]
        - Tear film [PMID 23769846, 23953715]
        - Energy reserve in copepods [PMID 32530916]
    - Wax diesters [FA0702]
        - Tear film [PMID 33954946]; Vernix caseosa [PMID 25555408]; Skin [PMID 949482, 3185122]
- FAHFA: Fatty Acyl Hydroxy Fatty Acids [FA0709]
    - FAHFA (2-, 3-, 5-, 9-, 12- & 15-hydroxy):
        - **$[M-H]^-$ → $[FA]^-$ & $[OH-FA]^-$**
        - Fungi [PMID 33243144]
        - Anti-inflammatory & antidiabetic lipokines in mammals [PMID 25303528, 33348554, 33372146, 34453998]
    - ω-hydroxy FAHFA:
        - **$[M-H]^-$ → $[FA]^-$ & $[Very-Long-Chain-OH-FA]^-$**
        - Also termed O-Acyl Hydroxy Fatty Acids (OAHFA)
            - Act as surfactants in specialized biofluids including tears, seminal plasma, sperm, & amniotic fluid [PMID 27180330, 28950169, 33348554]
    - α-hydroxy FAHFA (AAHFA):
        - **$[M-H]^-$ → $[Butyric\ acid]^-$**
        - Gut bacteria [PMID 33313490]
    - Aminoacyl FAHFA
        - Gly-FAHFA:
            - **$[M-H]^-$ → $[Gly]^-$ = $[74.0248]^-$ & $[M - Acyl-FA]^-$, $[Gly + CO-CH_2]^-$ = $[116.0353]^-$**

    - *Bacteroides thetaiotaomicron & Cryptophaga johnsonae* Gram-negative bacteria [PMID 1885525, 30367006]
- Lys-FAHFA:
    - **$[M+H]^+$ → $[Lys]^+$ = $[147.1128$ & $129.1022]^+$ & $[MH$ – acyl substituent on the OH-FA$]^+$**
    - *Pseudopedobacter saltans & Flavobacterium johnsoniae* Gram-negative soil bacteria [PMID 26175720]
- OH-Lys-FAHFA:
    - **$[M+H]^+$ → $[OH\text{-}Lys]^+$ = $[163.1077, 145.0972$ & $128.0706]^+$ & $[MH$ - acyl substituent on the OH-FA$]^+$**
        - Isobars: Lys-FAHFA(OH)
        - Isobar Resolution: MS^2
    - *Pseudopedobacter saltans & Flavobacterium johnsoniae,* Gram-negative soil bacteria [PMID 26175720]
- Gln-FAHFA:
    - **$[M\text{-}H]^-$ → $[Gln]^-$ = $[145.0619]^-$, [Ester-linked FA$]^-$**
    - *E. coli* Gram-negative bacteria [PMID 28854168]
- Orn-FAHFA:
    - **$[M+H]^+$ → $[Orn]^+$ = $[133.0972$ & $115.0866]^+$ & $[MH$ - acyl substituent on the OH-FA$]^+$**
    - **$[M\text{-}H]^-$ → $[Orn]^-$ = $[131.0826]^-$, [Ester-linked FA]- , & [Orn-FA$]^-$**
    - *Planctomycetes spp. , Agrobacteriumtume faciens, Burkholderia spp., & Rhizobium spp.* Gram-negative bacteria [PMID 21205018, 21707055, 22958119, 23995937, 25862689, 33469548]
- OH-Orn-FAHFA increases stress tolerance in *Rhizobium tropici* Gram-negative soil bacteria [PMID 21205018]:
    - **$[M\text{-}H]^-$ → $[OH\text{-}Orn]^-$ = $[147.0775]^-$, [Ester-linked FA$]^-$, & [OH-Orn-FA$]^-$**
        - Isobars: MeOrn-, DiMeOrn-, TriMe-Orn-FAHFA (all 3-OH FA) & Orn-FAHFA(OH)
        - Isobar Resolution: MS^2
    - MeOrn-FAHFA:
        - **$[M+H]^+$ → $[MeOrn]^+$ = $[147.1128$ & $129.1022]^+$, [MH – acyl substituent on the OH-FA$]^+$ & $[Orn - NH_2]^+$ = $[116.0706]^+$**
    - DiMeOrn-FAHFA:
        - **$[M+H]^+$ → $[DiMeOrn]^+$ = $[161.1285$ & $144.1020]^+$, [MH – acyl substituent on the OH-FA$]^+$ & $[Orn - NH_2]^+$ = $[116.0706]^+$**
    - TriMeOrn-FAHFA:
        - **$[M+H]^+$ → $[Orn - NH_2]^+$ = $[116.0706]^+$**
- Gly-Ser-Lipids [FA0802]
    - Gly-Ser-OH-FA

        - **[M+H]$^+$ → [Gly-OH-FA]$^+$ & [OH-FA – 2H_2O]$^+$**
        - Bacteroidetes spp. Gram-negative bacteria [PMID 29686782, 34500631]
    - Gly-Ser-Orn-OH-FA:
        - **[M+H]$^+$ → [MH - Orn]$^+$ & [Gly-Ser-Orn]$^+$ = [277.1506]$^+$**
        - Bacteroiddetes spp. Gram-negative bacteria [PMID 34500631]
    - Gly-Ser-FAHFA:
        - **[M-H]$^-$ → [Ester-linked FA]$^-$, [M - ester-linked FA]$^-$, [M – ester-linked-FA – CH_2O]-**
            - Isobar: e.g. Gly-Ser-FAHFA 32:0 = Gln-FAHFA(OH) 32:0
        - Isobar Resolution: MS^2
        - Gly-Ser FAHFA 32:0 known as **Flavolipin**
        - Gram-negative bacteria: *Flectobacillus major* [PMID 10760472] & *Bacteroidetes spp*. [PMID 28207237, 29686782, 32912852, 34500631, 35080445]
        - Outer membrane of *Bacteroidetes spp*. Gram-negative bacteria [PMID 35080445]
        - TLR2 ligands & Antimicrobial FAHFA
    - Gly-Ser-Orn-FAHFA
        - **[M+H]$^+$ → [MH – ester-linked FA]$^+$**
        - *Bacteroiddetes spp*. Gram-negative bacteria [PMID 34500631]
    - Gly-Ser-Orn-FAHFA(OH)
        - **[M+H]$^+$ → [MH – ester-linked OH-FA]$^+$**
        - Bacteroiddetes spp. Gram-negative bacteria [PMID 34500631]
    - Gly-Ser-FAHFA-P-DG
        - **[M-H]$^-$ → [P-DG]$^-$, [P-FA]$^-$ & [P-C_3H_5O]$^-$ = [152.9958]$^-$**
        - *P. gingivalis* Gram-negative bacteria [PMID 32912852]

- Glyco-OH-FA
    - Fungal Sophorolipids [FA1302]
    - Candida bombicola [PMID 8585609, 20738402]
    - Sophorolipid acids (SLA)
        - **[M-H]$^-$ →**
        - **[M+H]$^+$→ [MH – hexose]$^+$ = [MH -180.0634 & -162.0641]$^+$**
        - Sophorose-OH-FA
    - Acetyl-SLA

        - **[M-H]$^-$ →**
        - **[M+H]$^+$→ [MH – hexose]$^+$ = [MH -180.0634 & -162.0641]$^+$**
        - Acetyl-Sophorose-OH-FA
    - Acetyl-Sophorolipid Lactones (Ac-SLL)
        - **[M-H]$^-$ →**
        - **[M+H]$^+$→ [MH – hexose]$^+$ = [MH -180.0634 & -162.0641]$^+$ & [MH – AcetylGlucopyranose]$^+$ = MH -222.0740 & -204.0634]$^+$**
        - Acetyl-Sophorose-OH-FA
    - Di-Acetyl-SLA
        - **[M-H]$^-$ →**
        - **[M+H]$^+$→ [MH – hexose]$^+$ = [MH -180.0634 & -162.0641]$^+$**
        - DiAcetyl-Sophorose-OH-FA
    - Di-Acetyl-SLL
        - **[M-H]$^-$ →**
        - **[M+H]$^+$→ [MH – hexose]$^+$ & [MH – AcetylGlucopyranose]$^+$ =[MH -222.0740 & -204.0634]$^+$**
- Glyco-FAHFA
    - Lipid A variants:
        - **[M-H]$^-$ → [OH-FA substituent]$^-$ & [M -H - (FAHFA + C_2H_3N)]$^-$**
        - Di-OH-Acyl-P-Glucosamine-FAHFA-Glucosamine
        - Gram-negative *P. aeruginosa* [PMID 33625828]
    - Rhamnolipids
        - Rhamnosyl-OH-FA
        - Rhamnosyl & Di-Rhamnosyl 3-OH-FAHFA
            - **[M-H]$^-$ → [FA 10:1]$^-$ = [169.1234]$^-$ & [Rhamnose]$^-$ = [163.0612]$^-$**
            - **DiRhamose moiety = Rham+C_2H_2O =[205.07176]$^-$ & Rham+$C_4H_4O_2$ = [247.08233]$^-$**

Product	[M-H]⁻		Product	[M-H]⁻		Product	[M-H]⁻		Product	[M-H]⁻
C8	141.0921		C10-C8	311.2228		Rham-C8	305.1606		DiRham-C8	451.2187
C10	169.1234		C10-C9	325.2384		Rham-C9	319.1762		DiRham-C9	465.2343
C11	183.1391		C10-C10	339.2541		Rham-C10	333.1919		DiRham-C10	479.2500
C12	197.1547		C10-C11	353.2697		Rham-C11	347.2075		DiRham-C11	493.2656
C13	211.1704		C10-C12	367.2854		Rham-C12	361.2232		DiRham-C12	507.2813
C14	225.186		C10-C13	381.3010		Rham-C13	375.2388		DiRham-C13	521.2969
Rhamnose	163.0612		C10-C14	395.3167		Rham-C14	389.2545		DiRham-C14	535.3126

- Gram-negative bacteria [PMID 26879646, 29523151, 33146788]
- Gram-positive *Planococcus spp.* [PMID 32240926]
- Biosurfactant antimicrobials

- Alkyl-FAHFA
  - **$[M-H]^-$**
  - 9-FAHFA-Ethanesulfonic acids
  - Sponges (LMFA07020002)
- Glycolipid-FAHFA
  - FAHFA 34:0;O-TG:
    - **$[M+NH_4]^+$ → $[MH – FAHFA\ 34:0;O]^+$ = $[MH -538.4961]^+$ & $[MH - 16:0]^+$ = $[MH -256.2402]^+$**
    - Adipocytes [PMID 31056915]
- Glycopeptidolipids (GlycoPL)
  - C-Mycosides: hFA-deoxytalose(0 to 2 acetyls)-Phe-Thr-Ala-Alaninol-Rhamnose (0 to 3 methyls)
    - **$[M-H]^-$ → $[M – deoxytalose]^-$ & $[M – rhamnose]^-$**
    - **$[M+Cl]^-$**
    - Gram-positive mycobacteria [PMID 7484363, 15942014, 18723691, 23019158]

Glycerolipids (GL):

- Monoradylglycerols
- MG (Monoacyl Glycerols) [GL0101]:
  - **$[M+Cl]^-$**
  - **$[M+NH4]^+$ → $[FA – OH]^+$**

- MGe (Monoalkyl Glycerols):
  - **$[M+Cl]^-$**
  - **$[M+H]^+$ → $[Glycerol]^+$ = $[93.0546 \& 75.0441]^+$**
  - Sponge [PMID 7861942]; Protazoans [PMID 3430407, 6819334]
  - *Pneumocystis spp* fungi [PMID 9788237]
  - Desulfatibacillum alkenivorans Gram-negative bacteria [PMID 25724965]
  - CNS & Glioma [PMID 18164358, 34621897, 3662177)
- Glyco-MG:
  - MHMG (Monohexosyl-MG) [GL0401]:
    - **$[M+NH4]^+$ → $[MH - hexose]^+$ & $[MG - H_2O]^+$**
    - MHMG in plant chloroplasts [PMID 31337054], algae & cyanobacteria [PMID 34681936], Gram-positive *Clostridium spp* [PMID 31368115]
      - Isobars: MonoGlucosyl-MG in Gram-positive bacteria [PMID 28223392, 33627509, 31368115]
      - Isobar Resolution: Chromatography
  - DHMG (Dihexosyl-MG):
    - **$[M+NH4]^+$ → $[MH\text{-}dihexose]^+$ = $[MH -342.1162]^+$**
    - DHMG in plant chloroplasts [PMID 31337054]
      - Isobars: DiGlucosyl-MG & Galactosyl-glucosyl-MG in Gram-positive bacteria [PMID 24046864, 33627509]
      - Isobar Resolution: Chromatography
  - SQMG (Sulfoquinovosyl-MG)
    - **$[M\text{-}H]^-$ → $[Sulfoquinovose – H_2O]^-$ = $[225.0075]^-$, $[SO_3H]^-$ = $[80.9646]^-$ & $[FA]^-$**
    - **$[M+NH_4]^+$ → $[MG - H_2O]^+$ & $[MG -H_2O – (FA – H_2O)]^+$**
    - Oral commensal Gram-positive bacteria [PMID 33627509]
- Aminoacyl MG
  - MGTS (MG-Trimethylhomoserine) [GL0601]:
    - **$[M+H]^+$ → $[Tri\text{-}Me\text{-}HomoSer + Glycerol]^+$= $[236.1491]^+$**
    - Extra-plastidial betaine lipids in plants [PMID 31337054] & algae [PMID 25549757, 34057605]
    - Also quoted as Lyso-DGTS
      - Isobars: MG-Hydroxymethyl-trimethyl-alanine (MGTA)
      - Isobar Resolution: Chromatography
  - MGCC (MG-Carboxy-hydroxymethyl-choline):

        - **$[M+H]^+$ → $[Choline]^+$ = $[104.1070]^+$**
        - Extra-plastidial betaine lipids in plants
    - Ala-MG (Alanine-MG):
        - **$[M+H]^+$ → $[Ala]^+$ = $[90.0550]^+$**

- Diradylglycerols
    - Diacyl Glycerols (DG) [GL0201]:
        - DG:
            - **$[M+Cl]^-$**
            - **$[M+NH4]^+$ → $[MG-H_2O]^+$ & $[FA-H_2O]^+$**
        - CDP-DG:
            - **$[M-H]^-$ → $[M – cytidine]^-$ = $[M -243.0855]^-$, $[M - DG]^-$ & $[CDP - H_2O]^-$ = $[384.0003]^-$**
            - **$[M+H]^+$**
            - Gram-positive C. glutamicum, C striatum, & S. mitis [PMID 29724782, 32659445, 33954950]
            - Precursors of membrane lipoteichoic acids in Gram-positive bacteria [PMID 32659445, 33627509]
        - DG-AEP (DG-Aminoethylphosphonate)
            - **$[M-H]^-$**
            - *Ciliophora* protozoa [PMID 5091943]
            - Isobars: e.g. DG-AEP 32:0 = PEe 32:0
            - Isobar Resolution: MS^2: **DG-AEP → $[AEP]^-$ = $[122.00123]^-$; PEe → $[P Etahanolamine]^-$ = $[140.0113]^-$**
        - MAGE (Monoacylglycerol ethers = Alkyl-Acyl Glycerols) [GL0202]
            - **$[M+NH4]^+$ → $[MH\text{-}fatty alcohol]^+$**
            - **$[M+Cl]^-$ →**
            - Isobars: Alkenyl-Acyl Glycerols (MAGP) [GL0204]: (e.g. EAG 32:0 = DGp32:1)
            - Isobar Resolution: MS^2
            - *Pneumocystis spp* fungi [PMID 9788237]
    - MAGP (Alkenyl-Acyl Glycerols):
        - **$[M+Cl]^-$ →**
        - Gram-positive *Clostridium spp* [PMID 31368115, 33974975]
    - Hexosyl MAGE:
        - **$[M+Cl]^-$ → $[DG - H_2O]^-$ & $[DG - H_2O - (FA - H_2O)]^-$**

    - **$[M+NH4]^+$ → $[MH - (hexose - H_2O)]^+$ = $[MH -162.0528]^+$**
    - Rat brain [PMID 32891384]
- Sulfogalactosyl-MAGE (Seminolipids):
  - **$[M-H]^-$ → $[Sulfogalactose - H_2O]^-$ = $[241.0024]^-$**
  - Seminal fluid [PMID 27180330]
- Glyco-DG
  - MHDG (Monohexosyl-DG) [GL0501]:
    - **$[M+NH4]^+$ → $[MH - hexose]^+$ & $[MG - H_2O]^+$**
    - MHDG in plant chloroplasts [PMID 31337054], algae & cyanobacteria [PMID 34057605, 34681936]
      - MonoGlucosyl-DG in *Clostridia spp. & Bacillus licheniformis* Gram-positive bacteria are precursors of membrane lipoteichoic acids [PMID 24046864, 27317428, 30586545, 33627509, 33974975, 31368115, 34531870], Gram-positive clostridia spp [PMID 27317428]
      - Gram negative *Synechococcus spp.* [PMID 21630589]
      - Isobars: Glucosyl = Galactosyl = Mannosyl = Inositol
      - Isobar Resolution: Chromatography
    - MHDGea & MHDGee:
      - **$[M+NH_4]^+$ → $[MH – Hexose]^{+\ +}$ & $[MH - 162.0528 – (Fatty alcohol - H_2O)]^+$**
      - *Mycobacterium spp* [PMID 21321121]
    - PE-MHDG:
      - **$[M-H]^-$**
      - *Clostridium spp.* Gram-positive bacteria [PMID 31368115, 33974975]
    - MHDG;O (Arabidopsides) [GL0501]
      - **$[M+Cl]^-$**
  - DHDG (Dihexosyl-DG):
    - **$[M+NH4]^+$ → $[MH – dihexose]^+$ = $[MH -342.1162]^+$, $[MH - dihexose - FA]^+$ & $[FA]^+$**
    - DHDG in plant chloroplasts [PMID 31337054], algae & cyanobacteria [PMID 34057605, 34681936], nanovesicles from Gram-positive *Lactobacillus johnsonii* [PMID 34531870].
    - Gram negative *Synechococcus spp.* [PMID 21630589]
    - Isobars: DiGlucosyl-DG & Galactosyl-glucosyl-DG in *Clostridia spp. & Bacillus licheniformis* Gram-positive bacteria are precursors of membrane lipoteichoic acids [PMID 24046864, 27317428, 30586545, 33627509, 33974975, 31368115, 34531870], Gram-positive clostridia spp [PMID 27317428]; Staphylococcus epidermis [PMID29402937]

    - Isobar Resolution: Chromatography
- LTAP (Lipoteichoic Acid Primer = DHDG-GroP):
  - **$[M-H]^-$ → $[GroP]^-$ = [171.0059 & 152.9953]$^-$ & [Hex-GroP - $H_2O]^-$ = [315.0481]$^-$ [M -Ala]$^-$ = [M - 89.0477]$^-$ & [FA]$^-$**
  - Lipoteichoic acid primer in *Listeria, Streptococci, Bacilli, & Clostridia spp.* Gram-positive bacteria [PMID 19682249, 24046864, 27134729, 30586545, 33627509, 33974975]
- LTAP-Ala:
  - **$[M-H]^-$ → [Ala = 88.0404]$^-$, $[GroP]^-$ = [171.0059 & 152.9953]$^-$ & [FA]$^-$**
  - Alanyl-Lipoteichoic acid primer in Gram-positive bacteria [PMID 27134729, 30586545]
- LTAP-diAla:
  - **$[M-H]^-$ → $[Ala]^-$ = [88.0404]$^-$, $[GroP]^-$ = [152.9953]$^-$ & [FA]$^-$**
  - Di-Alanyl-Lipoteichoic acid primer in Gram-positive bacteria [PMID 27134729]
- Trihexosyl-DG & Tetrahexosyl-DG:
  - **$[M+Cl]^-$**
  - Gram-positive clostridia spp [PMID 27317428, 33974975]
- SQDG (Sulfoquinovosyl-DG):
  - **$[M-H]^-$ → [Sulfoquinovose – $H_2O]^-$ = [225.0075]$^-$, $[SO_3H]^-$ = [80.9646]$^-$ & [FA]$^-$**
  - **$[M+NH_4]^+$ → [DG - $H_2O]^+$ & [DG -H_2O – (FA – H_2O)]$^+$**
  - Algae & cyanobacteria [PMID 34057605, 346819360], Oral commensal Gram-positive bacteria [PMID 33627509]
  - Gram negative *Synechococcus spp.* [PMID 21630589]
- GlcA-DG (Glucuronosyl-DG)
  - GLcA-DG:
    - **$[M-H]^-$ → [M - (FA-H_2O)]$^-$ & [FA]$^-$**
    - Gram-positive *C. glutamicum & Mycobacterium smegmatis* [PMID 839936, 32659445, 33954950].
    - Gram-negative *Pseudomonas diminata* [PMID 405393].
    - Plants & fungi [PMID 34260857] & algae [PMID 34057605]
    - Activation of killer T-cells [PMID 34123306]
  - Mannosyl-GlcA-DG:
    - **$[M-H]^-$ → [M – mannose]$^-$ = [M -180.0634]$^-$ & [FA]$^-$**
    - Gram-positive C. glutamicum [PMID 32659445, 33954950]
- Glucosaminyl-DG
  - **$[M-H]^-$ → [DG FAs]$^-$, [M-FA]$^-$ & [GP – $H_2O]^-$ = [152.9958]$^-$**

        - *Bacillus megaterium* Gram-positive bacteria [PMID 4627584]
    - GlcNAc-DG:
        - GlcNAc-DG (N-Acetylglucosamine-DG):
            - **$[M+Cl]^-$**
            - **$[M+H]^+$ → $[MH$ - glucuronic acid$]^+$ = $[MH -176.0321]^+$**
            - Gram-positive *Clostridia spp.* [PMID 20173213, 22837302]
            - Accumulates with phosphate deprivation in plants & Algae [PMID 27023231, 30786236]
        - PE-GlcNAc-DG:
            - **$[M-H]^-$ → $[PE]^-$ = $[140.0113]^-$, $[M – DG]^-$, $[M – sn2\ FA]^-$ & $[FA]^-$**
            - Gram-positive *Clostridia spp.* [PMID 20173213, 22837302, 33974975]
        - GlcNAc-DGpa (N-Acetylglucosamine-Alkenyl-Acyl-DG):
            - **$[M+Cl]^-$**

c) Aminoacyl DG:

- DGTS (DG-Trimethylhomoserine) [GL0501]:
    - **$[M+H]^+$ → [TriMeHSer-Glycerol$]^+$ = $[236.1491]^+$,$[MH-(FA - H_2O)]^+$ & [TriMeHSer$]^+$ = [162.1125 & 144.1010$]^+$**
    - **$[M+Cl]^-$ → [M -N-Trimethyl$]^-$ = $[M- 59.0735]^-$, $[FA]^-$ & $[C_4H_5O_2]^-$ = $[83.0139]^-$**
    - Extra-plastidial betaine lipids in nonvascular plants [PMID 31337054], soil bacteria, fungi & algae [PMID 8892811, 24728191, 25549757, 25724745, 26895707, 34057605]
    - Replaces membrane phosphatidylcholine in fungi [PMID 24728191], algae [PMID 29555786], and *Agrobacterium tumefaciens*, a Gram-negative plant pathogen [PMID 229581] experiencing limited phosphate supplies
        - Isobars: DG-Hydroxymethyl-trimethyl-alanine (DGTA)
        - Isobar Resolution: Chromatography
    - Algae [PMID 25549757, 26895707]
- DGCC (DG-Carboxy-hydroxymethyl-choline) [GL0701]:
    - **$[M+H]^+$ → [Choline = 104.1070$]^+$ & [CMCh – CO_2 = 132.1019$]^+$**
    - Extra-plastidial betaine lipids in plants & fungi [PMID 9375419] & algae [PMID 26895707]
- Ala-DG (Alanine-DG):
    - **$[M+H]^+$ → $[Al]^+$ = $[90.0550]^+$ & $[DG-H_2O]^+$**
    - *C. glutamicum & Bacillus subtilis* Gram-positive bacteria (PMID 26235234, 27134729, 29724782)
- Lys-DG:
    - **$[M-H]^-$**

        - Gram-positive *Staphylococcus spp.* [PMID29402937]
    - Lys-Gal-DG:
        - **$[M-H]^-$ → $[Lys]^-$ = $[145.0983]^-$ & $[FA]^-$**
        - **$[M+H]^+$ → $[Gal-Lys]^+$ = $[291.1551\ \&\ 273.1445]^+$, $[Lys-H_2O]^+$ & $[C_4H_5NO]^+$ = $[84.0444]^+$**
        - Gram-positive Streptococcus spp. [PMID 35180210]
        - Highly positively charged. Involved in bacteria crossing BBB.

- Triradylglycerols
    - TG (Triacyl Glycerols) [GL0301]:
        - **$[M+NH_4]^+$**
    - DAGE (Diacyl-Glycerol Ether = Alkyl-Acyl-Acyl TG) & AGdiE (Acyl-glycerol diether) [GL0302]:
        - **$[M+H]^+$ → $[MH-FA]^+$ & $[MH\text{-Fatty alcohol}]^+$**
            - Isobars: DG (e.g. DAGE 40:0 = DG 40:0)
            - Isobar Resolution: MS^2
        - Gram-negative bacteria: *Sorangium cellulosum* & *Desulfatibacillum alkenivorans* [PMID 21321121, 25724965] & *Myxococcus xanthus* [PMID 16990257]
        - *Dermabacter hominis Gram-positive bacteria [PMID 26867985]*
        - Coral [PMID 34711899]; Sea cucumber [PMID 35877727]; Pteropods & Copepods [PMID 19493709]
    - TG-OH-Acyl (Estolides) [GL0305]:
        - **$[M+NH_4]^+$**
        - In plant oils and adipose tissue functioning as a metabolite reservoir of FAHFAs that are released by lipases [PMID 33372146]

Glycerophospholipids (GPL)

- Glycerophosphoglycerols
    - PG (Diacyl-GPG) [GP0401]:
        - **$[M-H]^-$ & $[M+NH_4]^+$**
        - Gram-positive bacteria [PMID 28223392], skin & fibroblasts [PMID 20945919]
        - High levels & diversity in krill oil [PMID 21593706, 33669109]
            - Isobars: Acyl-lyso-PG (A-LPG) also annotated as Lyso-bisphosphatidic acids (LBPA) or BMP [GP0410]
            - Isobar Resolution: MS^2
        - PG present in human tears [PMID 24287121],

        - **$[M-H]^-$ → [FA2 >FA1]- for both PG & A-LPG**
        - **$[M+NH_4]^+$ → $[DG \& (DG-H_2O)]^+$ for PG**
        - **$[M+NH_4]^+$ → $[MG \& (MG-H_2O)]^+$ for A-LPG**
- LPG (Lyso-PG) [GP0405]
    - **$[M-H]^-$ → $[FA]^-$**
- PGp (Alkenyl-Acyl-PG; plasmalogens) [GP0403]
    - **$[M-H]^-$**
    - Gram-positive *Clostridium spp* [PMID 31368115, 33974975]
- PGP (PG-glycerophosphate):
    - **$[M-H]^-$ & $[M+NH_4]^+$**
- PG precursor [PMID 34901881]
- Acyl-PG:
    - **$[M-H]^-$ → $[FA]^-$, $[M - sn2\ FA]^-$, & $[M - acyl\ FA]^-$**
    - **$[M+NH_4]^+$ → $[DG-H_2O]^+$ & $[MG-H_2O]^+$; $[M-H]^-$ → $[acyl\ FA]^-$**
    - 3 acyl chains; Also termed semilysobisphosphatidic acid (SLBPA)
    - Gram-positive *C. glutamicum* [PMID 29724782]
    - Palmitoyl-PG in Gram-negative Bacteria: *E. coli* [PMID 30660747], *Salmonella typhimurium* [PMID 14698549, 24449881], *Klebsiella pneumoniae* [PMID 35841948], & *Actinetobacter spp.* [PMID 29748546]
- PE-Acyl-PG:
    - **$[M-H]^-$ → $[FA]^-$, $[M - sn2\ FA]^-$, $[PA]^-$, & $[Phosphoethanolamine]^-$ = $[140.0114]^-$**
    - Gram-negative *Actinetobacter spp.* [PMID 29748546]
- CL (Cardiolipins; Tetraacyl-PG) [GP0201]:
    - **$[M-H]^-$ → $[FA]^-$ & $[PA]^-$**
- Sulfo-PG:
    - PG-Sulfate:
        - **$[M-H]^-$**
        - *Archaea* [PMID 12923225, 19054744, 20538644]
    - Sulfohexosyl PG
        - **[M-H] →**
        - *Archaea* [PMID 26*134137]*
- PG-GroP-Me (Ptd-glycerophosphate methyl ester)

        - **[M-H] →**
        - *Archaea* [PMID 12923225, 30930858*]*
- Ceramide-PG (Ceramide phosphorylglycerol)
    - **[M-H]⁻ → [P-Glycerol]⁻ = [171.0064 & 152.9958]⁻**
    - Abundant membrane lipid in Gram-negative bacteria *P. gingivalis, Bacteroides fragilis* involved in periodontal disease [PMID 15466368, 21347306, 31067435, 35877858] & *Flectobacillus major* [PMID 10760472]
        - Isobars: Cer-PG;O3 = PSp; Cer-PG;O2 = PE
        - Isobar Resolution: MS^2
- Aminoacyl-PG
    - Ala-PG:
        - **$[M+H]^+$ → $[Ala]^+$ = $[90.0550]^+$ & $[DG / (DG\text{-}H_2O)]^+$**
        - **$[M\text{-}H]^-$ → $[Ala]^-$ =[88.0404$]^-$ & $[FA2 > FA1]^-$**
        - *B. subtilis, C. glutamicum, P. aeruginosa, Bifidobateria spp. & Clostridia spp*. Gram-positive bacteria [PMID 19787708, 21195206, 22837302, 26235234, 26998233, 27134729, 29724782]
    - Leu-PG:
        - **$[M\text{-}H]^-$ → $[Leu]^-$ = $[130.0974]^-$**
        - *B. subtilis* Gram-positive bacteria [PMID 26998233, 27134729]
        - Isobars: Leu-PG 38:3 = Amadori-PEp 38:4
        - Isobar Resolution: MS^2**: Leu-PG →$[Leu]^-$; Amadori-PEp → $[M - sugar]^-$ = $[M – 162.0528]^-$** [PMID 33111151]
    - Lysyl-PG: **$[M\text{-}H]^-$ → $[Lys]^-$ = $[145.0983]^-$ & $[FA2 > FA1]^-$**
        - *Bacillus spp., Cohnella spp., Enterococcus spp., Lactobacillus spp., Staphylococcus spp., Listeria spp., Streptococcus spp., C. glutamicum, & Clostridia spp*. Gram-positive bacteria [PMID 19787708, 21195206, 22837302, 26235234, 26998233, 27134729, 27317428, 27506207, 28423018, 29402937, 29724782, 30586545]
    - Succinyl-Lys-PG:
        - **$[M\text{-}H]^-$ → $[Succ\text{-}Lys]^-$ = $[245.1143 \& 227.1037]^-$, $[Lys]^-$ = $[145.0983]^-$ & $[Lysyl\text{-}PG]^-$**
        - *B. subtilis* Gram-positive bacteria [PMID 27134729, 27506207]
    - Arg-PG:
        - **$[M+H]^+$ → $[Arg]^+$ = $[175.1190 \& 157.1084]^+$**
        - *Enterococcus faecium* Gram-positive bacteria [PMID 19787708]
    - Asp-PG:
        - **$[M\text{-}H]^-$ → $[Asp]^-$ = $[132.0302 \& 114.0197]^-$**

        - *B. subtilis* Gram-positive bacteria [PMID 26998233, 27134729]
    - Orn-PG:
        - **[M+H]$^+$ → [Orn]$^+$ = [133.0972 & 115.0866]$^+$**
        - *Mycobacteria spp.* & Gram-positive *Bacillus cereus* [PMID 19787708, 5414606, 19787708]
- Glyco-PG
    - Glucosaminyl-PG
        - **[M-H]$^-$ → [PG FAs]$^-$, [M – FA]$^-$, & [GP – H_2O]$^-$ = [152.9958]$^-$**
        - *Bacillus megaterium* Gram-positive bacteria [PMID 4309309, 4975666]
        - *Pseudomonas putida* [PMID 5964949] & *P. aeruginosa* [PMID 30171632] Gram-negative bacteria.

➢ Glycerophosphocholines (GPC)

- PC (Diacyl-GPC) [GP0101]:
    - **[M+H]$^+$ → [P-choline]$^+$ = [184.0739]$^+$**
    - **[M+Cl]$^-$ → [FA2 > FA1]$^-$**
- PCe (Alkyl-Acyl-GPC) [GP0103]
    - **[M+H]$^+$ → [P-choline]$^+$ = [184.0739]$^+$**
    - **[M+Cl]$^-$ → [FA2 > FA1]$^-$**
- PEp (Alkenyl-Acyl-GPC; plasmalogen) [GP0105]
    - **[M+H]$^+$ → [P-choline]$^+$ = [184.0739]$^+$**
    - **[M+Cl]$^-$ → [FA2 > FA1]$^-$**
- Lyso-PC
    - **[M+H]$^+$ → [P-choline]$^+$ = [184.0739]$^+$**
    - **[M+Cl]$^-$ → [FA2 > FA1]$^-$**
- LPCp:
    - **[M+H]$^+$ → [P-choline]$^+$ = [184.0739]$^+$, [MH – alkenyl fatty alcohol]$^+$ & [PC – Quat N]$^+$ = [125.0004]$^+$**
    - **[M+Cl]$^-$ → [M – CH_3]$^-$ = [M - 15.0235]$^-$, [P-Choline – CH_3]$^-$= [168.0431]$^-$, [GP]$^-$ = [152.9958]$^-$ & [Fatty alcohol -2H]$^-$**

➢ Glycerophosphoethanolamines (GPE)

- PE (Diacyl-GPE) [GO0201]:
    - **[M-H]$^-$ → [FA2 > FA1]$^-$ & [PE headgroup]$^-$ = [140.0118]$^-$**
    - **[M+H]$^+$ → [MG+H -H_2O]$^+$**
    - High levels & diversity in krill oil [PMID 21593706, 33669109]
- NAPE (N-Acyl-PE):

- **[M-H]$^-$ → [PE-FA]$^-$, [M-H - N-Acyl-FA]$^-$ & [P-NAE = N-Acyl FA + PE headgroup = N-Acyl FA + 140.0113]$^-$**
    - *Actinetobacter spp.* Gram negative bacteria [PMID 29748546]
- PEe (Alkyl-Acyl-GPE) [GP0202]
    - **[M-H]$^-$ → [FA2 > FA1]$^-$ & [PE headgroup]$^-$ = [140.0118]$^-$**
- N-Acyl-PEp (NAPEp)
    - **M-H]$^-$ → [PE-FA]$^-$, [M-H - N-Acyl-FA]$^-$ & [P-NAE = N-Acyl FA + PE headgroup = N-Acyl FA + 140.0113]$^-$**
- PEp (Alkenyl-Acyl-GPE, plasmalogen) [GP0203]:
    - **[M-H]$^-$ → [FA2 > FA1]$^-$ & [PE headgroup]$^-$ = [140.0118]$^-$**
    - **[M+H]$^+$ → [MG+H -H_2O]$^+$**
- GA-PEp (Glycerol acetal PEp):
    - **[M-H]$^-$**
    - *Clostridium spp.* Gram-positive bacteria [PMID 26746573, 2837302, 31368115, 33974975]
- PG-GA-PEp
    - **[M-H]$^-$**
    - *Clostridium spp.* Gram-positive bacteria [PMID 22837302, 31368115, 33974975]
- LPE (Lyso-PE):
    - **[M-H]$^-$ → [FA]$^-$ & [GPE – H_2O]$^-$ = [196.0375]$^-$**
    - **[M+H]$^+$ → [MG+H -H_2O]$^+$**
- LPEe:
    - **[M-H]$^-$ → [PE headgroup]$^+$ = [140.0118]$^-$**
    - **[M+H]$^+$ → [MH – PE]$^+$ = [MH - 141.0191]$^+$ & [PE]$^+$ = [142.0263]$^+$**
- LPEp:
    - **[M-H]$^-$ → [PE headgroup]$^-$ = [140.0118]$^-$, [GPE - H_2O]$^-$ = [196.0375]$^-$ & [Alkenyl fatty alcohol]$^-$**
- Acyl-PE:
    - **[M+NH_4]$^+$ → [DG - H_2O]$^+$, [PA]$^+$, & [FA-Amide]$^+$**
    - Gram-negative *E. coli* [PMID 30660747]
- Aminoacyl-PE
    - Ala-PE
        - **[MH]$^+$ → [DG – H2O]$^+$ & [Alanyl-ethanolamine - H_2O]$^+$ = [115.0866]$^+$**
        - *Bacillus subtilis* Gram-positive bacteria [PMID 26998233]
    - Lysyl-PE

        - **[MH]$^+$ → [DG – H_2O]$^+$, [Lys - H_2O]$^+$ = [129.1022]$^+$ & [Lysyl-phosphoethanolamine]$^=$ = [270.1213]$^+$**
        - *Bacillus subtilis* Gram-positive bacteria [PMID 26998233]
- Ceramide-PE (Ceramide phosphorylethanolamine) [SP0302]:
    - **[M-H]$^-$ → [PE]$^-$ = [140.0113]$^-$**
    - **[MH]$^+$ → [MH – PE]$^+$ = [MH -141.01909 & 159.0297]$^+$, [PE]$^+$ = [142.0263]$^+$ & [d-sphingolipid base -$2H_2O$]$^+$**
    - Abundant membrane lipid in Gram-negative bacteria (*P. gingivalis, Tannerella forsythia, Bacteroides fragilis, B. thetaiotaomicron*) involved in periodontal & intestinal homeostasis , & in invertebrates & parasites [PMID 15466368, 21347306, 31067435, 31071294, 35080445, 35877858]
        - Isobars: SM & CAEP: (e.g. SM 30:0;O2 = CerPE 33:0;O2 = CAEP 33:0;O3)
        - Isobar Resolution: Both lipid families form [M+H]$^+$ ions in PES., In contrast, in NESI, SMs form chloride adducts [M+Cl]$^-$ and Cer-PE form [M-H]$^-$ ions. MS^2 analysis is also valuable.
- OXPE (Oxidized PE)
    - Amadori PE, PEe, PEp, LPE [GP21]
        - **[M-H]$^-$ → [M - sugar]$^-$ = [M – 162.0528]$^-$ & [FA]$^-$**
        - Human plasma Amadori products [PMID 33111151]
    - PE carboxyalkylpyrroles (CEP, CPP, CHP)
        - **[M-H]$^-$**
        - Angiogenic factors in retina & tumors [PMID 23847393, 25380349], Hyperlipidemic mice [PMID 27015965], Astrocytes & microglia in ALS [PMID 34871763]

➢ Glycerophosphoserines (GPS)

- PS (Diacyl-GPS) [GP0301]:
    - **[M-H]$^-$ → [PA]$^-$ & [FA2 > FA1]$^-$**
    - **[M+H]$^+$ → [MH – Ser headgroup]$^+$ = [MH - 185.0089]$^+$**
- NAPS (N-Acyl-PS):
    - **[M-H]$^-$ → [PA]$^-$ & [FA2 > FA1]$^-$**
    - Augmented in the frontal cortex in schizophrenia [PMID 26120595, 31434624]
    - Human retina & optic nerve [PMID 22266369]
- PSe (Alkyl-Acyl-GPS):
    - **[M-H]$^-$ → [ether PA]$^-$, [ether LPA]$^-$ & [FA]$^-$**
    - Decrements in frontal cortex in MCI & AD [PMID 36004004]
- PSp (Alkenyl-Acyl (Plasmalogen):

        - **[M-H]$^-$ → [vinylether PA]$^-$, [vinylether LPA]$^-$ & [FA]$^-$**
        - Human retina & optic nerve [PMID 22266369]
    - LPS (Lyso-GPS)
        - **[M-H]$^-$ → [FA]$^-$**

➢ Glycerophosphoinositols (GPI)

- PI (Diacyl-GPS) [GP0401]:
    - **[M-H]$^-$ → [FA]$^-$ & [PI headgroup – H_2O]$^-$ = [241.0113]$^-$**
    - **[M+H] → [MH – PI headgroup]$^+$ = [MH -260.0293]$^+$**
- LPI (Lyso-PI) [GP0405]
    - **[M-H]$^-$ → [FA]$^-$ & [PI headgroup – H_2O]$^-$ = [241.0113]$^-$**
- Glyco-GPI: Mannosyl-PIs (PIMs) are unique to *Actinobacteria* Gram-positive bacteria (e.g. *Mycobacteria spp. & C. glutamicum*) [PMID 25862689, 27114527, 27887882]
    - PIM1 (Mannosyl-PI) [GP1501]:
        - **[M-H]$^-$**
        - **[M+NH_4]$^+$**
        - Gram-positive *C. glutamicum* [PMID 29724782]
    - LPIM1 (Lyso-PIM1) :
        - **[M+NH_4]$^+$**
    - Acyl-PIM1:
        - **[M+NH_4]$^+$**
    - PIM2 (Di-Mannosyl-PI1):
        - **[M+NH_4]$^+$**
        - Gram-positive *C. glutamicum* [PMID 29724782] & *Mycobacterium bovis* [PMID12753185]
    - PIM3 (Tri-Mannosyl-PI):
        - **[M+NH_4]$^+$**
    - Lyso-PIM3:
        - **[M+NH_4]$^+$**
    - Lyso-PIM4 [SP0303]:
        - **[M+NH_4]$^+$**
- Ceramide-PI
    - Biosynthesis: DHC → Phytoceramide & OH-Phytocer → IPC → MIPC → M(IP)2C

        - Plants [PMID 32961698], Fungi [19174513, 30206958, 16498600, 32033944], Gram-negative *Bacteriodes spp*. Outer membrane vesicles [PMID 35080445]
    - IPC;O2, O3, & O4 (Ceramide-PI) [SP0303]
        - **[M-H]$^-$**
        - Gram-negative *Bacteriodes thetaiotaomicron* [PMID 35080445]
    - GIPC (GlcN-GlcUA-PI-Ceramide):
        - Hexose:
            - **[M-H]$^-$ → [IP]$^-$ = [259.0220 &241.0113]$^-$ & [IP – GlcA – Hexose]$^-$ = [597.1069]$^-$.**
            - **[M+H]$^+$ → [t-Sphingolipid base – 2H_2O]$^+$ & [Cer xy;O4 - H2O]$^+$**
            - Fungi [PMID 17971386, 25309884]
        - Aminohexose:
            - **[M-H]$^-$ → [IP]$^-$ = [259.0220 &241.0113]$^-$ & [IP – GlcA – AminoHexose]$^-$ = [596.1229]$^-$**
            - **[M+H]$^+$ → [t-Sphingolipid base – 2H_2O]$^+$ & [Cer xy;O4 - H2O]$^+$**
            - Fungi [PMID 17971386, 25309884]
        - N-Acetyl-Aminohexose:
            - **[M-H]$^-$ → [IP]$^-$ = [259.0220 &241.0113]$^-$ & [IP – GlcA – Ac-AminoHexose]$^-$ = 638.1334]$^-$**
            - **[M+H]$^+$ → [t-Sphingolipid base – 2H_2O]$^+$ & [Cer xy;O4 - H2O]$^+$**
            - Most abundant sphingolipid in plants & fungi [PMID 23887274, 27074617, 32961698]
    - MIPC;O2, O3 & O4 (Mannosyl-Ceramide-PI)
        - **[M-H]$^-$**
    - M(IP)2C;O2, O3 & O4 (Mannosyl-Ceramide-PI2)
        - **[M-H]$^-$**

- Glycerophosphoalkylamines:
    - **[M-H]$^-$**
    - PGLN (Diacyl-PA-Acyl-alkylamine-Glucosamine)
    - PGL (Diacyl-PA-Acyl-alkylamine-N-Acetyl-Glucosamine)
        - Gram-negative *Thermus* bacteria [PMID 22815675, 36345289]
- PA (Phosphatidic acids)
    - PA [GP1001]:
        - **[M-H]$^-$ → [M – H – FA]$^-$, [FA]$^-$ & [GP]$^-$ = [152.9953]$^-$**
        - Gram-positive bacteria [PMID 28223392]

- PAe:
  - **[M-H]$^-$ → [PO_3]$^-$= [78.9585]$^-$**
- LPA:
  - **[M-H]$^-$ → [GP]$^-$ = [152.9953]$^-$**
- Cyclic PA (cPA):
  - **[M-H]$^-$ → [FA]$^-$**
- PGlu (Phosphatidylglucoside)
  - **[M-H]$^-$**
  - Gram-positive Staphylococcus aureus (PMID 5473881) & Gram-negative Mycoplasma laidlawii (PMID 4297044)
  - Outer leaflet of plasma membrane with sterols in bacteria & mammals (PMID 20433166, 22227109)
  - Biomarker of human neural stem cells (PMID 21306383)
- PAHN (Phosphatidyl-N-AcetylHexosamine)
  - **[M-H]$^-$ → [M – FA]$^-$, [FA]$^-$, & [M – (NAcHex – H_2O)]$^-$ = [M- 203.07937]$^-$**
  - Gram-positive bacteria *S. mitis* [PMID 28223392]

Sphingolipids

- Ceramides
  - Cer;O (Deoxyceramides) [SP0206]:
    - **M+H]$^+$ → [M -Sphingosine base – H_2O]$^+$**
    - Alanine replaces serine in biosynthetic pathway, hence the loss of a hydroxy group
    - Critical scaffold lipids in antigen-presenting CD1 system [PMID 22087000]
    - Only m18:1 forms in mouse CNS; accumulate in spinal cord & sciatic nerve with age [PMID 30790665]
    - Both m18:0 & m18:1 in human plasma [PMID 27388466]
    - Deoxyceramides stimulate autophagy in mouse embryonic fibroblasts [PMID 32835606]
    - Accumulate in serum & visceral adipose tissue in Type 2 diabetes [PMID 33351229]
  - Cer;O2 (Ceramides & Dihydroceramides) [SP0201 & SP0202]:
    - **[M+Cl]$^-$ → [M-H]$^-$**
    - **[M+H]$^+$ → [d-Sphingosine base – $2H_2O$]$^+$**
    - Skin ceramides [PMID 32265320]

- Cer;O3 (Phytoceramides) [SP0203]:
    - **$[M+Cl]^-$ → $[M-H]^-$**
    - **$[M+H]^+$ → [t-Sphingosine base – $2H_2O]^+$**
    - Skin ceramides [PMID 32265320]
- Cer;O4 (OH-Phytoceramides):
    - **$[M+Cl]^-$ → $[M-H]^-$**
    - **$[M+H]^+$ → [t-Sphingosine base – $2H_2O]^+$**
- Cer;O5 (Di-OH-Phytoceramides) [SP0204]:
    - **$[M+Cl]^-$ → $[M-H]^-$**
    - **$[M+H]^+$ → [t-Sphingosine base – $2H_2O]^+$**
- ω-Linoleoyloxy Phytoceramides:
    - **$[M+Cl]^-$ → $[M-H]^-$**
    - **$[M+H]^+$ → [t-Sphingosine base – $2H_2O]^+$**
- Ceramide sulfates:
    - Sulfonolipids
    - **$[M-H]^-$**
    - *Bacteroidetes* [PMID 36952261]
    - Sulfatides (SulfoHexyosyl-Cer;O2)
    - **$[M-H]^-$**
    - OH-Sulfatides
    - **$[M-H]^-$**
- Glycoceramides
    - Hexosyl-Cer (GlcCer):
        - **$[M+H]^+$ → [Sphingosine base – $2H_2O]^+$ & [MH – hexose$]^+$ = [MH - 180.0634 & -162.0528$]^+$**
        - Glucosyl/galactosyl ceramides in fungi [PMID 25309884]
    - Hexosyl-OH-Cer
    -
    - Hexosyl-Phytocer
    -
    - Hexosyl-DHC
    -

    - Di-Hexosyl-DHC
        - **[M+H]$^+$ → [Sphingosine base – 2H_2O]$^+$ & [MH – dihexose]$^+$ = [MH -342.1162]$^+$**
- CAEP (Ceramide aminoethylphosphonates)
    - **[M-H]$^-$ → [FA]$^-$, [AEP]$^-$ = [124.0169]$^-$, [M - N-Acyl FA]$^-$ & [M - FA]$^-$**
    - **[M+H]$^+$ → [AEP]$^+$ = [126.0315]$^+$,[MH – AEP]$^=$ = [MH -125.02418 & -143.03474]$^+$ & [MH – AEP – N-acyl FA]$^+$**
    - Isobars: SM & Cer-PE (e.g. SM 30:0;O2 = CerPE 33:0;O2 = CAEP 33:0;O3)
    - Molluscs [PMID 31067435, 31476549, 34711899]
- Sphingolipid bases
    - Sphingolipid base variants
    - Sphingolipid base glycosides
    - Sphingofungins (Guanidino-modified base)
- Acyl Ceramides
    - Acyl-Cer
    - Skin Acyl-Cer
- Sulfonolipids:
    - O-Acyl halocapnines
        - **[M-H]$^-$ → [Halocapnine - H_2O]$^-$ = [418.2269]$^-$, [Halocapnine - H_2O - H_2SO_4]$^-$= [336.2544]$^-$ & [FA or OH-FA]$^-$**
        - *Salisaeta longa* halophilic Gram-negative bacteria of *Bacteroidetes* branch [PMID 20211932]

➢ Sphingomyelins
- SM
    - **[M+H]$^+$ = [P-choline]$^+$ = [184.0739]$^+$**
    - **[M+Cl]$^-$ = [FA]$^-$**
    - Isobars: CerPE & CAEP (e.g. SM 30:0;O2 = CerPE 33:0;O2 = CAEP 33:0;O3)
- OH-SM
-
- Lyso-SM (LSM)
-

➢ Gangliosides
- Gb3 series (Hex3Cer;O2)
    - **[M-H]$^-$ → [Neuraminic acid - H_2O]$^-$ = [249.089]$^-$**
- GM3 (NeuAcHex2Cer;O2)

- **[M-H]$^-$ → [Neuraminic acid - H_2O]$^-$ = [249.089]$^-$**

Isopentyl-PP Products

- Sterols (ST)
  - Cholesterol & similar sterols
    - Cholesterol [ST0101]:
      - **[MH-H_2O]$^+$ = [369.3516]$^+$**
      - Major membrane sterol in mammals & in copepods [PMID 25092258]
    - Cholesterol glucoside:
      - **[M+NH_4]$^+$= [566.4415]$^+$ → [Cholesterol -H_2O]$^+$= [369 .3516]$^+$**
      - **[M+Cl]$^-$ = [583.3775]$^-$**
      - Steryl glycosides play a role in evading host immune responses [PMID 25195783]
    - CAG (Cholesteryl Acyl-α-glycosides): Cholesterol-Hexose-DAG
      - **[M-H]$^-$ → [M – Cholesterol]$^-$ = [M - 386.3487]$^-$ & [FA]$^-$**
      - **[M+NH_4]$^+$ →**
      - Extremely hydrophobic esters
      - *Helicobacter pylori* & *Borrelia burgdorferi* Gram-negative bacteria [PMID 21635569, 7665522, 32991669, 34061884]
      - Secretion by *H. pylori* protects against host lysosomal degradation of bacteria [PMID 34706729]
      - In fungi steryl-β-glucosides are critical membrane constituents (e.g. ergosteryl-glucoside in *Cryptococcus neoformans*; [PMID 25361768])
    - CE (Cholesterol esters):
      - **[M+NH_4]$^+$**
      - Gram-positive Staphylococcus spp. [PMID 32554713]
    - CS (Cholesterol sulfate):
      - **[M-H]$^-$ → [H_2SO_4]$^-$ = [96.9596]$^-$**
    - CPG (Cholesteryl Acyl-phosphoglycosides): Cholesterol-P-Hexose-DG
      - **[M-H]$^-$ → [M – Cholesterol]$^-$ = [M- 386.3487]$^-$ & [FA]$^-$**

- **$[M+NH_4]^+$ → $[DG - H_2O]^+$**
- *Helicobacter pylori* & *Borrelia burgdorferi* Gram-negative bacteria [PMID 7665522, 32991669, 34061884]

- CEPG (Cholesteryl P-Ethanolamine Glycoside): Cholesterol-Hexose-P-Ethanolamine
  - **$[M+H = 672.4235]^+$ → $[Cholesterol - H_2O]^+ = [369.3516]^+$, $[P\text{-}Ethanolamine]^+ = [142.0264]^+$ & $[Hexosyl\text{-}PE]^+ = [304.0792]^+$**
  - *Helicobacter pylori* Gram-negative bacteria [PMID 32991669]
- Ergosterol
  - **$[M+H]^+ = [397.3465]^+$ →**
  - Major sterol in fungal membranes [PMID 36354897]
- Ergosteryl glucoside
  - **$[M+Cl]^- = [593.3619]^-$**
  - Fungal membranes [PMID 26322039, 36354897]

- Bactoprenols
  - Bacterial hopanoids [PR04]
    - Hopanoids:
      - **$[M+H]^+$ →**
      - Pentacyclic triterpinoids that provide membrane stability in place of cholesterol
      - *Burkholderia spp., Acetobacter spp., & Cyanobacteria spp.* Gram-negative bacteria, except *P. aeruginosa* which lacks hopanoids [PMID 17294511, 24957830, 31499020] & *Planctomycetota* [PMID 25862689]
      - Lichens, plants & bacteria [PMID 29456243]
    - Hopanetetrol cyclitol ethers:
      - **$[M+H]^+$ → $[C_8H_{10}NO_6]^+ = [222.0972]^+$ & $[C_6H_{13}NO_5]^+ = [180.0867]^+$**
      - Gram-negative bacteria [PMID 24957830]
  - Isoprenoids [PR 01]
    - IPP (isoprenoid diphosphates):
      - **$[M-H]^-$ → $[PO_3 = 78.9585]^-$ & $[P_2O_6H_2 = 158.9254]^-$**
    - Undecaprenol (C55) [PR0301]
      - C55-P:
        - **$[M-H]^-$**
        - **$[M+NH_4]^+$**
        - Gram-positive S. mitis [PMID 3291669],
    - C55-PP [PR0303]

        - C55-PP-Galactose [PR0303]
        - C55-PP-GlcNAc-Man-NAcA
        - Type IV LTA intermediates
            - Gram-positive oral commensal bacteria [PMID 33627509]
                - C55-PP-AATGal (C55-PP-Acetamido-Amino-Deoxygalactopyranose; CID 90658310)
                - C55-PPAAT-Gal-Gal
                - C55-PP-Gal-Gal-Ribose-P
- Polyketides
    - PM (Phosphomyoketides)
    - MPI (Mannosyl-P-isoprenoids):
        - **$[M\text{-}H]^-$ → $[M\text{-}H_2O]^-$ & $[M - (Mannose - H_2O)]^-$**
        - Gram-positive *Mycobacteria spp*. [PMID 15611286]
- Isoprenyl PG
- Archaebacterial Modified Isoprenyl PG
    - PG-Phosphonate-Me-Ester (Me-PGP)
    - Sulfotrehalose-Galactosyl-PG (STG-PG)
- Carotenoids
    - Carotenoids & OH-Carotenoids [PR0107]:
        - **$[M+H]^+$ → $[Toluene - H_2]^+$ = $[91.0542]^+$, $[Me\text{-}Toluene - H_2]^+$ = $[105.0855]^+$, $[Di\text{-}Me\text{-}Toluene - H_2]^+$ = $[119.0855]^+$ & $[MH - toluene]^+$ = $[MH - 92.0526]^+$**
        - Carotenoids are secondary metabolites derived from isoprenoid metabolism
        - Carotenoids are organic pigments synthesized from lipid precursors by algae, bacteria, fungi, and plants [PMID 16001255, 31284598, 3294126]. The long hydrocarbon chains of these pigments span membranes with the terminal ring groups residing at the interior and exterior surfaces of membranes. These terminal ring groups act as antioxidants at the membrane surfaces.
        - Gram-negative marine bacteria [PMID 24663119] & microalgae [PMID 31284598]
        - Carotenoid esters: **$[M+H]^+$ → $[MH - FA]^+$**
        - Glyco-Carotenoids
        - Fungal Apocarotenoids
- Algal isoprenyls
- Chlorophylls:
    - **$[M\text{-}H]^-$ → $[M - 76.0160]^-$**

    - **$[M+H]^+$ → $[MH - phytyl\ group]^+ = [MH -278.2974]^+$ & $[MH-C_2H_4O_3]^+ = [MH -76.0160]^+$**
    - Plants [PMID 21905735, 34940601]
- Copepodamides (Isoprenyl-Taurine)
    - Copepodeamides ($-CH_3$ & $=CH_2$ families):
        - **$[M-H]^-$ → $[Taurine]^- = [124.0074]^-$, $[C_{22}H_{43/41}NO_5S]^- = [432.2789/430.2633]^-$ & $[SO_3]^- = [79.9568]^-$**
        - Copepodamides are polar isoprenoid fatty acids with a terminal amide linkage to the amino acid taurine. These amino lipids are produced and released into the water solely by copepods where they stimulate toxin production by phytoplankton to evade predation [PMID 25918403].

Secondary Metabolites

- Marine bryostatins
- Bacterial diterpinoids
    - Tuberculosinol:
        - **$[M+H]^+$**
        - Diterpinoid virulence factor of Gram-positive *Actinobacteria*
    - Tuberculosinyladenosine:
        - **$[M+H]^+$ → $[C_5H_5N_5]^+ = [136.0618]^+$ & $[C_{25}H_{37}N_5]^+ = [408.3122]^+$**
    - Algal bromo-diterpinoids: **$[M-H]^-$**

Environmental Biomarkers

- Perfluoroalkyl pollutants:
    - **$[M-H]^-$ → $[SO_3]^- = [79.9568]^-$**
    - Human cord blood [PMID 28883994]
- D-Acyl-Chloropropanediols
- Glycidyl FA Esters
- Mycotoxins
